# Multiresponsive Cylindrically Symmetric Cholesteric Liquid Crystal Elastomer Fibers Templated by Tubular Confinement

**DOI:** 10.1002/advs.202301414

**Published:** 2023-04-26

**Authors:** Yong Geng, Jan P.F. Lagerwall

**Affiliations:** ^1^ Experimental Soft Matter Physics group Department of Physics and Materials Science University of Luxembourg L‐1511 Luxembourg Luxembourg

**Keywords:** cholesteric liquid crystal elastomer fibers, cylindrical symmetry, mechanochromism, soft actuators, thermochromism, tubular confinement

## Abstract

Cylindrically symmetric cholesteric liquid crystal elastomer (CLCE) fibers templated by tubular confinement are reported, displaying mechanochromic, thermochromic, and thermomechanical responses. The synthesis inside a sacrificial tube secures radial orientation of the cholesteric helix, and the ground state retroreflection wavelength is easily tuned throughout the visible spectrum or into the near‐infrared by varying the concentration of a chiral dopant. The fibers display continuous, repeatable, and quantitatively predictable mechanochromic response, reaching a blue shift of more than −220 nm for 180% elongation. The cylindrical symmetry renders the response identical in all directions perpendicular to the fiber axis, making them exceptionally useful for monitoring complex strains, as demonstrated in revealing local strain during tying of different knots. The CLCE reflection color can be revealed with high contrast against any background by taking advantage of the circularly polarized reflection. Upon heating, the fibers respond—fully reversibly—with red shift and radial expansion/axial contraction. However, there is no transition to an isotropic state, confirming a largely forgotten theoretical prediction by de Gennes. These fibers and the easy way of making them may open new windows for large‐scale application in advanced wearable technology and beyond.

## Graphical abstract

Cylindrically symmetric cholesteric liquid crystal elastomer fibers are synthesized, revealing mechanochromic, thermochromic, and thermomechanical responsiveness with diverse application potential, for example, in smart responsive textiles.

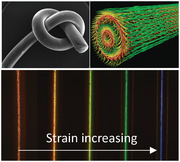

## 1 Introduction

Mechano‐ and/or thermochromic elastomeric fibers, changing color in response to strain and temperature change, respectively, can be very useful for strain and temperature sensing in, for example, health care, sports, or safety gear, where the light‐weight soft and flexible form factor and independence of power sources gives advantages with respect to alternative technologies.^[^ ^1^, ^2^, ^3^, ^4^, ^5^ ^]^ They can also enable entirely new possibilities in fashion and artistic design.^[^ ^6^, ^7^ ^]^ The strongest mechanochromic response is found in structurally colored rubbers,^[^ ^8^ ^]^ exhibiting a periodic modulation of the refractive index that couples to the rubber network, such that mechanical strain changes the periodicity with consequent color change. The periodic modulation can be created top‐down by imprinting photosensitive rubbers^[^ ^9^ ^]^ or stacking sheets with different refractive indices,^[^ ^10^, ^11^ ^]^ or bottom‐up using liquid crystal self‐assembly, yielding cholesteric liquid crystal elastomers (CLCEs).^[^ ^12^, ^13^, ^14^, ^15^ ^]^ We focus on the latter approach, building on recent results on CLCE fibers that demonstrate strong potential for responsive garments.^[^ ^15^ ^]^

The structural color of CLCEs is due to two simultaneous spontaneous ordering processes: at the molecular scale, the rod‐like molecules locally self‐assemble along a common direction called the director, **n**, and on the scale of about a micron, **n** rotates in a helical fashion along an axis **m** perpendicular to **n**.^[^ ^16^, ^17^ ^]^ The molecular scale self‐assembly leads to optical anisotropy with greater local refractive index along **n** than perpendicular to it (or vice versa), such that the rotation of **n** results in a corresponding helical modulation of the effective refractive index. Since the helix pitch *p* is often on the order of visible light wavelengths and is easily adjustable by varying the chemical composition, the CLCE becomes a colorful Bragg reflector active in a narrow wavelength range that can be tuned throughout the visible spectrum and into the near‐infrared (IR) or near‐ultraviolet (UV) regions.

The wavelength λ of maximum reflection intensity, as measured in air, is given by Bragg's law

(1) $$\lambda =\bar{n}p\cos\theta =\lambda_{0}\cos\theta$$

where $\bar{n}$ is the average refractive index in the CLCE and *θ* is the angle of incidence between the illuminating light ray and **m**. For *θ* = 0, that is, in retroreflection configuration, the reflected wavelength has its maximum value $\lambda_{0}=\bar{n}p$. We use λ_0_ as a reference parameter to compare the reflection characteristics of different CLCEs at varying strain. As is clear from Equation (1), control of *p* and **m** (determining *θ*) are critical for obtaining the operation desired from a CLCE. In case of fibers, the ideal geometry is cylindrical with radial **m**, the rubber elasticity converting any tensile strain $\varepsilon_{zz}=\frac{\Delta L}{L^{*}}>0$ along the fiber (the $\hat{z}$‐direction; the original fiber length is $L^{*}$ and the length change Δ*L*) into a compression of the radius *r* and thus of *p*, giving a blue shift of λ_0_ away from its relaxed‐state maximum value $\lambda_{0}^{*}$.^[^ ^15^ ^]^

The Bragg reflection takes place symmetrically around the λ given by Equation (1) over a bandwidth equal to

(2) $$\Delta \lambda =p\Delta n_{\text{nh}}$$

where Δ*n*_nh_ is the birefringence in the absence of helix (nh: non‐helical). A decrease in *p*, as expected if ϵ_*zz*_ > 0, thus leads not only to a reduction in λ_0_ but also to a narrowing of the reflection band.

We recently produced mechanochromic CLCE fibers by depositing an extruded oligomeric CLCE precursor solution on a rotating mandrel, photocrosslinking after annealing into a CLCE.^[^ ^15^ ^]^ While we could demonstrate fast, reversible, and continuous mechanochromic blue shift, the production method yields a fiber cross section that was belt‐like rather than cylindrical, and the delicate balance of simultaneous processes taking place prior to crosslinking renders the procedure sensitive to any deviation from optimum processing parameters. Moreover, it is challenging to scale up the mandrel‐based process to match industrial expectations on yield.

Here we overcome these limitations by synthesizing the CLCE fiber inside the core of a low‐density polyethylene (LDPE) tube that can easily be dissolved after the process is complete. In addition to templating a cylindrical shape and preventing any break‐up triggered by capillary forces, the confinement within a tube that promotes tangential **n** at the tube wall enforces the desired radial alignment of **m** as the cholesteric structure of the precursor liquid crystal develops.^[^ ^18^, ^19^, ^20^ ^]^ We achieve cylindrical CLCE fibers of unprecedented quality with radial **m** and excellent control of *p*, exhibiting highly attractive mechanochromic, thermochromic, and thermomechanical responses.

## 2 Results and Discussion

### 2.1 Fabrication of CLCE Fibers and Characterization of Relaxed‐State Structure

**Figure 1:**
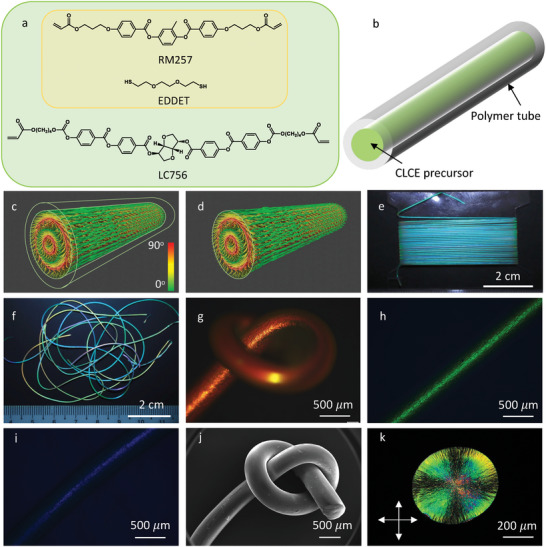
CLCE fiber production via LDPE tube confinement. a) Reactive liquid crystal monomer RM257 and flexible chain extender EDDET (yellow box) react to form nematic oligomers, prior to tube filling or within the tube, and the chiral dopant LC756 is added to turn the phase cholesteric. b) The CLCE precursor, either a solution of oligomer, chiral dopant, and dichloromethane (DCM) or a melt of the monomer mixture, is injected into an LDPE tube with 0.5–0.6 mm inner diameter. c,d) Schematic drawings of the cholesteric order with radial helix developing within the tube (c; color represents the orientation of **n** with respect to the fiber axis, as indicated in the color legend) and (d) after crosslinking and tube removal. e,f) Macroscopic views of (e) 2.5 m of CLCE fiber with blue‐green $\lambda_{0}^{*}$ collected on a flat winder, and (f) of several fibers with red, green and blue $\lambda_{0}^{*}$, respectively, randomly placed on a black table. g–i) Reflection POM (crossed polarizers) images of CLCE fibers with green and blue $\lambda_{0}^{*}$, respectively. j) SEM image of a knot in a fiber with red $\lambda_{0}^{*}$, shown in reflection POM in (g). k) A transmission POM (crossed polarizers) image of a 5 *µ*m thick CLCE fiber cross‐section slice.

In a first approach, we prepare reactive main‐chain non‐chiral nematic LC oligomer via self‐limiting thiol‐acrylate Michael‐addition reaction,^[^ ^21^ ^]^ linking the diacrylate mesogenic monomer RM257 with the dithiol chain extender EDDET (see **Figure** **1**a for chemical structures). In our previous work,^[^ ^15^ ^]^ we measured $\bar{M_{n}}\approx 10.8$ kg mol^−1^ for oligomers synthesized in the same way. Each oligomer contains on the average fourteen RM257 residues separated by EDDET residues, and it is terminated at both ends by acrylate groups. These will eventually be photopolymerized to turn the system into a soft elastomer with low crosslink density. We dissolve the oligomer in DCM along with the reactive chiral dopant LC756 (Figure 1a) and photo‐initiator, filling the solution into an LDPE tube (b). A minimum concentration of DCM (20wt%, giving a solution that is isotropic at room temperature and at rest) is required in this approach to lower the viscosity to a range where the solution can easily be filled into the tube.

After filling, the tube is placed in an incubator at 45 °C for the DCM to diffuse through the tube wall and evaporate over the course of 2 days (LDPE is permeable to DCM), allowing the CLC phase to develop, with **m** oriented radially (Figure 1c). Importantly, the CLCE precursor in the tube is in a liquid state capable of flowing and the DCM diffusion through the LDPE softens the tube wall, allowing capillary forces between the liquid interior and the tube wall to ensure that the core is completely filled throughout the process. There is thus no risk of wall detachment, void formation or Rayleigh–Plateau instability.

Once the cylindrically symmetric cholesteric order is stable, the precursor is crosslinked by UV irradiation through the tube wall. To minimize heating from the long and high‐power UV exposure, the tube is submerged into a water bath. Afterward, the LDPE tube is dissolved by immersing in toluene at a temperature of 90–100°C, kept inside a closed vessel for 30 min. Being a cross‐linked polymer network at this stage, the CLCE core cannot be dissolved, hence this process removes only the LDPE tube, releasing the finished CLCE fiber to be harvested. After rinsing three times in fresh toluene to remove any residual LDPE, and then evaporating all toluene in a vacuum oven at 150°C for 2 h, the CLCE fiber is ready to characterize and use. Its radial helix alignment, now permanented through the rubber network and not requiring any confining tube as support, is schematically illustrated in Figure 1d.

Since the applied pressure required to actively fill a tube scales linearly with viscosity and tube length according to the Hagen–Poiseuille equation, the viscosity of the oligomer solution is challenging if long tubes are used. As an alternative approach, we thus fill the tubes with the molten mixture of the original monomers, having significantly lower viscosity than the oligomer solution. Both the first step Michael‐addition reaction into oligomers and the second step UV‐induced cross‐linking will then take place inside the tube. Using a syringe pump, we can easily fill tubes of several meters length with this melt, and the CLCE synthesis works equally well as when starting with an oligomer solution. An example of a 2.5 m long CLCE fiber synthesized in this way is shown in Figure 1e. When synthesizing this fiber, the catalyst was diluted in a small amount of DCM for better controlling its concentration, but this is not required. We have repeatedly obtained equally good results with pure catalyst added to the monomer melt, thus allowing fully solvent‐free CLCE synthesis in the tubular template.

By changing the concentration of the chiral dopant, we easily tune $\lambda_{0}^{*}$ from IR to blue as desired, as shown in Figure 1f where several fibers with different $\lambda_{0}^{*}$ are randomly placed together on a black background and photographed using a regular DSLR camera. Panels (g)–(i) show reflection polarizing optical microscopy (POM) images of fibers with red, green, and blue $\lambda_{0}^{*}$, respectively. Our production method ensures that the fibers adopt the cylindrical morphology with uniform diameter and smooth surface of the interior of the LDPE tube from which they are templated, as shown in Figure 1j, showing the same fiber as in panel (g) in which a knot has been tied near the end, imaged by scanning electron microscopy (SEM). Note that the lower depth of field of POM renders the fiber end sticking out of the knot almost invisible in (g).

To confirm the radial helix alignment at microscopic scale, we first cut a 5 *µ*m thick cross section slice from a fiber using a microtome and investigate it in POM, see Figure 1k and Figures S1 and S2, Supporting Information. The Maltese cross in Figure 1k is the classic POM fingerprint of a birefringent sample with radial optic axis. As explained in Section S2, Supporting Information, this is what we expect for radial **m** and *p* short enough to give visible Bragg reflection. The pattern extends throughout the cross section, indicating excellent radial helix uniformity. We investigate the helix orientation also on macroscopic scale, by studying the retroreflection color along the full length of a fiber as it is viewed along different radii, see Figure S3 and Section S3, Supporting Information. The constant appearance gives additional proof of radial **m** throughout the fiber, constituting a major advance from the earlier belt‐shaped fibers.^[^ ^15^ ^]^

### 2.2 Mechanochromic Response

Although CLCEs are anisotropic,^[^ ^14^ ^]^ the cylindrical symmetry invites to analyze the mechanochromic response considering a single Poisson's ratio *ν* that relates the strain ϵ_*rr*_ along the radial direction $\hat{r}$ (thus along the helix axis **m**) to the strain ϵ_*zz*_ along the fiber axis $\hat{z}$. Neglecting variations in *ν* upon strain, we can then use the standard mechanical analysis of cylindrical rods under large tensile strain

(3) $${\left(1+\varepsilon_{zz}\right)}^{-\nu }=\left(1+\varepsilon_{rr}\right)=\left(1+\frac{\Delta r}{r^{*}}\right)=\frac{r}{r^{*}}=\frac{p}{p^{*}}=\frac{\lambda_{0}}{\lambda_{0}^{*}}$$

where the asterisk superscript indicates parameter values in the relaxed state. We can thus fit a function

(4) $$\lambda_{0}=\lambda_{0}^{*}{\left(1+\varepsilon_{zz}\right)}^{-\nu }$$

to obtain our Poisson's ratio of interest directly from the mechanochromic data. Our choice to follow the cylindrical symmetry convention with $\hat{z}$ along the cylinder axis means that, when a Cartesian coordinate system is used, this will be rotated 90° compared to most theoretical treatments of CLCEs,^[^ ^22^, ^23^, ^24^, ^25^ ^]^ which generally considered flat films with **m** along $\hat{z}$ and tensile strain along $\hat{x}$.

**Figure 2:**
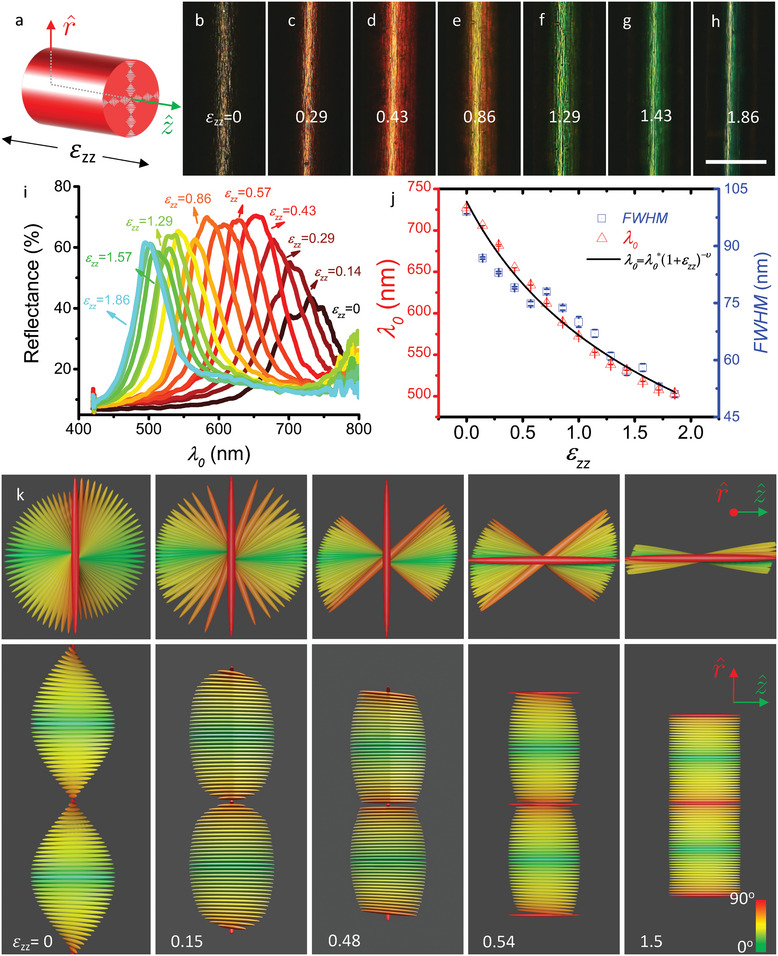
Mechanochromic response of CLCE fibers. a) Schematic fiber section for defining our cylindrical coordinate system. b–h) Reflection‐mode micrographs (obtained without polarizers) of a fiber with near‐IR $\lambda_{0}^{*}$ under gradually increasing elongational strain ϵ_*zz*_ (scale bar in h is 0.5 mm). i) Reflection spectra along $\hat{r}$ obtained without polarizers, corresponding to (bh). j) Retroreflection wavelength λ_0_ and full width at half maximum (FWHM) of spectra in i) versus ϵ_*zz*_. The black line is the best fit of Equation (4). k) Schematic depictions of strain‐induced deformation and eventually unwinding of the cholesteric helix, viewed along (upper row) and perpendicular to (lower row) the helix axis **m** $\left|\right|\hat{r}$. The color coding reflects the orientation of each rod (extending from −**n** to +**n**) in the undistorted helix (ϵ_*zz*_ = 0), green $\left|\right|\hat{z}$, and red $⊥\hat{z}$. Each rod retains the same color in all images in order that its reorientation can easily be tracked.

We show a qualitative demonstration of the mechanochromic response in Movie S1, Supporting Information, for four fibers simultaneously. From bottom to top, they exhibit $\lambda_{0}^{*}$ in the near‐IR, red, green, and blue. The reflectance of the bottom fiber is measured as a function of ϵ_*zz*_, see **Figure** **2**, where the coordinates with respect to the fiber geometry are defined in panel (a). The corresponding data for fibers with red and yellow $\lambda_{0}^{*}$, respectively, are shown in Figures S4 and S5, Supporting Information. Results from mechanical testing, including stress–strain cycling, rupture tests and determination of Young's modulus, are shown in Figure S6, Supporting Information. In the images in Figure 2b–h (reflection microscopy without polarizers) we see that the color blueshifts gradually as the fiber is stretched, although at ϵ_*zz*_ = 0 and 1.86, respectively, the fiber appearance is dominated by specular reflection at the fiber–air interface. A more accurate view of the Bragg reflection at all strain levels is provided by the reflection spectra, also obtained without polarizers but within a smaller region of the fiber (about 10 *µ*m diameter), shown in panel (i).

The spectra are fitted with a Lorentz function to establish λ_0_ and the full width at half maximum (FWHM) as a function of ϵ_*zz*_, see Figure 2j. Both parameters decrease with increasing strain, following the same trend. This is consistent with previous studies^[^ ^23^, ^25^ ^]^ and is expected from Equation (2), predicting a bandwidth that scales with *p*: as λ_0_ decreases due to compressed *p*, FWHM should decrease in the same manner, as indeed seen. We see a maximum blueshift Δλ_0_ ≈ −223 nm from the original $\lambda_{0}^{*}\approx 726$ nm to λ_0_ ≈ 503 nm at ϵ_*zz*_ = 1.8. This is significantly stronger shift than with our previous CLCE fibers, where we measured Δλ_0_ ≈ −155 nm shift at ϵ_*zz*_ = 2. With flat film or ribbon CLCEs, as investigated previously, the contraction $⊥\hat{z}$ is shared between $\hat{x}\left|\right|$ **m** and $\hat{y}⊥$ **m**. In contrast, in the present fibers with true cylindrical symmetry, contraction is always along **m**. We believe this may be the explanation of the stronger color shift, as discussed further in Section S4, Supporting Information.

Fitting Equation (4) to the data in Figure 2j, we obtain $\lambda_{0}^{*}=741\pm 4$ nm and *ν* = 0.37 ± 0.008. Interestingly, ν increases with decreasing $\lambda_{0}^{*}$, fibers with red and yellow $\lambda_{0}^{*}$ yielding *ν* ≈ 0.42 and *ν* ≈ 0.50, respectively (see Figure S5, Supporting Information). This suggests that the mechanical behavior of the CLCE approaches that of an isotropic rubber (*ν* ^iso^ = 0.5) as $p^{*}$ decreases. The increasing Poisson's ratio is highly valuable from the perspective of applying the CLCE fibers as strain sensors in garments, since it leads to a greater relative wavelength shift, in particular for small strains. As shown in Section S4, Supporting Information, a change from *ν* = 2/7—typical for flat CLCE sheets and the ribbon‐shaped fibers in our previous work—to *ν* = 0.5 results in a 73% increase in relative wavelength shift induced by a small strain of ϵ_*zz*_ = 0.1.

The maximum reflection intensity increases from ≈45% in the relaxed fiber to ≈70% for ϵ_*zz*_ ≈ 0.5, then gradually decreases to ≈60% for ϵ_*zz*_ ≈ 2. This phenomenon, where a stretched CLCE has higher reflectance of unpolarized light than the 50% maximum of a relaxed cholesteric (it reflects only the circular polarization with the handedness of the helix), has been observed before.^[^ ^26^, ^27^ ^]^ To understand the origin we illustrate in Figure 2k the “local optic axis” orientation (each rod extends from −**n** to +**n**) along one helix pitch *p* at different strain levels, calculated using Warner–Terentjev theory,^[^ ^22^, ^25^ ^]^ see Section S6, Supporting Information (Figure S7 and S8, Supporting Information). These drawings should not be understood as representing the entire fiber cross section, but rather a very small local region near the fiber perimeter, within which **m** can be approximated as constant. As a consequence, the radial direction $\hat{r}$ is approximated as constant, out of the image plane in the upper row and upward in the image plane in the lower row. The stretching direction $\hat{z}$ is to the right in all images. An additional viewing direction is shown in Figure S9, Supporting Information, and in Figure S10, Supporting Information, the director field at each strain level is drawn replicated along the radii of a circular cross section. We find that the strain not only compresses *p*, explaining the blueshift of λ_0_, but the helix also gets “flattened” into the *rz*‐plane as *ϵ*_*zz*_ keeps increasing. Regions with **n** $\left|\right|\hat{z}$ expand while the twist gets increasingly localized within *π* rotation walls around points where $n⊥\hat{z}$. Optically, we increasingly lose the chiral structure selecting one circular polarization over the other, approaching that of a traditional multilayer structure with higher reflectivity at the cost of reduced polarization contrast. Beyond a critical strain $\varepsilon_{zz}^{c}$, the *π* walls are unwound and **n** is no longer perpendicular to $\hat{z}$ at any point. This reduces the contrast in index modulation, which would explain the decreased reflectivity upon higher strain. We therefore do the modeling for Figure 2k such that $\varepsilon_{zz}^{c}\approx 0.5$, yielding maximum reflectance in (i).

### 2.3 Thermochromic and Thermomechanical Response

LCEs as well as densely crosslinked glassy LC networks (gLCNs) are well known for their mechanical actuation, changing shape in response to temperature changes.^[^ ^28^ ^]^ The classic LCE actuation is an entropically driven shape change related to the polymeric network changing conformation from anisotropic to isotropic upon heating through the LC–isotropic transition,^[^ ^29^ ^]^ thus with maximum response near the transition temperature *T*_c_. This transition does not exist in gLCNs, due to their much stronger degree of crosslinking, and they therefore actuate continuously as a result of anisotropic thermal expansion.^[^ ^28^ ^]^ In both cases, the material contracts along **n** and expands in the perpendicular plane on heating. Most LCE actuation work has been done on non‐chiral LCEs, but in his seminal theoretical paper on the order of polymer networks formed in a liquid crystal environment, and how this should change in case the environment becomes isotropic, Pierre‐Gilles de Gennes actually considered also cholesterics.^[^ ^30^ ^]^ He noted that if a network is formed in the helically modulated director field of a cholesteric, the polymer network cannot relax to an isotropic configuration: the cholesteric‐templated network is topologically constrained to retain the helical configuration with at least some degree of order, in contrast to the case of networks formed in non‐chiral nematics or smectics.

This suggests that no LC–isotropic transition should exist in CLCEs, even if the degree of crosslinking is much lower than in gLCNs. Consequently, the common route to distinguish between LCEs and gLCNs by testing whether a clearing point exists needs to be revised if also cholesteric LCEs are considered. In the absence of a clearing point, we cannot expect that CLCEs will show the entropically driven actuation localized mainly to a specific temperature range that is characteristic of non‐chiral LCEs. Qualitatively, we might rather expect a thermomechanical behavior of our CLCE fibers that is closer to that of gLCNs. For flat sheets of cholesteric gLCN, Broer and Mol found significant thermal expansion along **m** upon heating, while in the plane ⊥**m** a compression of much smaller magnitude was measured.^[^ ^31^ ^]^ The strong thermal expansion along **m** redshifts $\lambda_{0}^{*}$, as has been demonstrated also for CLCEs by several groups, studying sheets and ribbons^[^ ^26^, ^27^, ^32^, ^33^, ^34^ ^]^ or spheres.^[^ ^35^ ^]^ To the best of our knowledge, however, nobody has deliberately tested de Gennes' theoretical prediction of the loss of the LC–isotropic transition in CLCEs. While some experimental papers report a phase transition in CLCEs that might appear as a clearing point, this cannot be the case for the global 3D CLCE since the Bragg reflection color remains at all temperatures. Rather, there may be local clearing in small pockets that are disconnected from the global network, because only a very sparse first‐stage crosslinking was carried out prior to detecting the transition,^[^ ^26^ ^]^ and/or because the CLCE was pre‐stretched between the first‐stage sparse crosslinking and final photocrosslinking,^[^ ^26^, ^27^ ^]^ thus partially unwinding the helix and thereby creating local regions in which the topological constraints that prevent the clearing transition are not formed.

**Figure 3:**
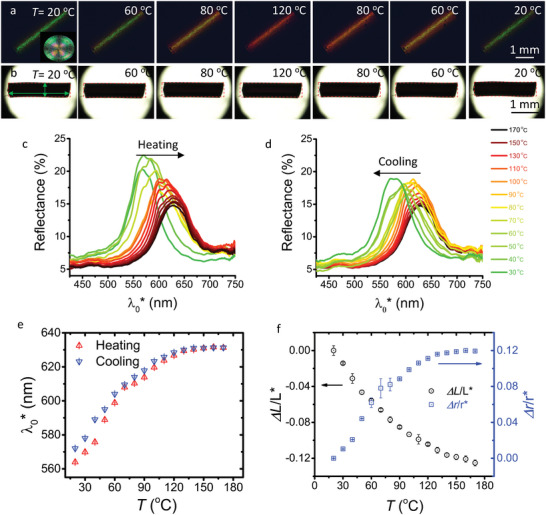
Thermochromic and thermomechanical response of CLCE fibers. a,b) Reflection‐ and transmission‐mode POM images of a piece of CLCE fiber with green $\lambda_{0}^{*}$ at room temperature during heating and cooling. The orange box in (b) traces the boundaries at 20 °C. c,d) Retroreflection spectra (without polarizer) corresponding to (a) during heating and cooling. e) Unstrained retroreflection wavelength $\lambda_{0}^{*}$ during heating and cooling. f) Relative relaxed‐state length and radius changes, Δ*L*/$L^{*}$ and Δ*r*/$r^{*}$, respectively, as function of temperature.

Given the radial orientation of **m**, we expect that our CLCE fibers subject to heating should redshift and expand along $\hat{r}$, while they should contract along the fiber length. As summarized in **Figure** **3**, we study the color and shape of a fiber section that at room temperature has length $L^{*}$ ≈ 2.25 mm, radius $r^{*}$ ≈ 0.26 mm, and green $\lambda_{0}^{*}\approx 565$ nm. We confirm that the fiber exhibits the desired radial **m** from POM of a fiber cross‐section (inset in Figure 3a with full details in Section S2, Supporting Information). Upon heating from 20 to 170 °C, we take reflection photos of the fiber at regular intervals, as shown in Figure 3a (more images can be found in Figure S11, Supporting Information), and in a subsequent repetition the fiber shape and dimensions are monitored in transmission (Figure 3b). During the reflection photography experiment we also acquire reflection spectra, see Figure 3c,d. Panel (e) shows $\lambda_{0}^{*}$ as function of temperature and panel (f) shows the relative length and radius changes (they are plotted as function of each other in Figure S12, Supporting Information). The radius change is obtained optically as $\frac{\Delta r}{r^{*}}=$ $\frac{\lambda_{0}^{*}\left(T\right)-\lambda_{0}^{*}\left(20^{\circ }C\right)}{\lambda_{0}^{*}\left(20^{\circ }C\right)}$, thus assuming that $p^{*}$ scales with $r^{*}$ and that $\bar{n}$ does not change significantly.

As the fiber is heated, we confirm the expected redshift, from $\lambda_{0}^{*}\approx 565$ nm to $\lambda_{0}^{*}\approx 630$ nm at 120 °C (see also Movies S2–S3, Supporting Information). For higher temperatures the curve flattens out, and the color remains red over 120–170 °C. Figure 3c reveals that the reflection peak weakens and widens upon heating. The former suggests some reduction in order while the latter reflects the increase in $p^{*}$ according to Equation (2). We see in (f) that $r^{*}$ expands and $L^{*}$ contracts, as expected, but in contrast to the flat gLCN sheet studied by Broer and Mol, our contraction and expansion have similar magnitude. While the cylindrical geometry of our fibers may have an impact on the different response, we believe it is primarily the lower degree of crosslinking, rendering our fibers true elastomers, that allows stronger thermomechanical actuation ⊥**m** than in flat gLCE sheets.

Confirming de Gennes' prediction, we see no sign of a clearing transition, as the fibers remain colored up to 170 °C (Figure S11, Supporting Information) and as no transition above the glass transition *T*_g_ is detected in differential scanning calorimetry (DSC), see Figure S13, Supporting Information. For comparison, our chemistry is a chiral modification of the original chemistry developed by Yakacki et al. to make non‐chiral nematic LCEs, showing classic entropic LCE actuation in the range 40–60 °C in the absence of externally imposed strain.^[^ ^36^ ^]^ The absence of a clearing transition makes the CLCE fibers thermodynamically comparable to gLCNs above *T*_g_, and we thus attribute the monotonic actuation seen over the entire temperature range studied to anisotropic thermal expansion, as in gLCN actuation.

### 2.4 Application Demonstration

#### 2.4.1 Revealing the Mechanochromic Strain Sensing Response without Background Using Circular Polarizers

The soft cylindrical form factor, high elasticity and machine washability^[^ ^15^ ^]^ render our mechanochromic CLCE fibers ideal for incorporation as non‐electronic visual‐feedback strain sensors in smart clothing or other textile products like tents, furniture or sails. However, in a visually busy environment, the CLCE fibers may be difficult to detect from the background. To reveal them regardless of how the carrier fabric is colored or patterned, we can take advantage of the circular polarization of CLCE reflections, following a concept recently demonstrated using polymerized cholesteric spheres.^[^ ^37^, ^38^, ^39^ ^]^

**Figure 4:**
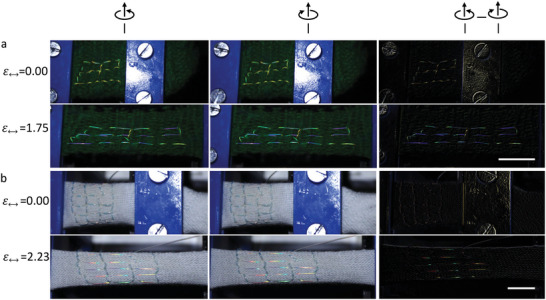
Enhanced visualization of mechanochromic response by circular polarization‐enabled background subtraction. By photographing a textile with incorporated CLCE fiber through a right‐handed (left column) and left‐handed (middle column) circular polarizer, respectively, and subtracting the latter from the former, the background is removed while the CLCE reflection remains visible with enhanced contrast (right column). a) A fiber with green $\lambda_{0}^{*}$ is sewn in a grid pattern into a loose wool fabric with dark green color and then photographed in the relaxed (top row) and stretched state (second row). b) The same experiment with a fiber with near‐IR $\lambda_{0}^{*}$ sewn into a white stretchy fabric. (Scale bars are 1 cm).

**Figure** **4** shows two CLCE fibers, with green (a) and near‐IR $\lambda_{0}^{*}$ (b), sewn into fabrics with green and white color, respectively. The samples are photographed in the relaxed state (ϵ_↔_= 0) and during horizontal strain of the carrier fabric (ϵ_↔_ > 0), through a right‐handed (left column) and through a left‐handed circular polarizer (middle column). Because the background is unpolarized, it appears identical in the two images, but the CLCE fiber appears slightly more intense through right‐ than through left‐handed polarizer due to its circularly polarized reflection. The difference is so small that it is difficult to notice by simply comparing the two columns. However, by subtracting the middle from the left column, even the subtle intensity difference becomes strikingly visible in the relaxed as well as the strained states for the fiber with green $\lambda_{0}^{*}$ since the background is deleted by this action, as demonstrated in the right column. The same applies to the fiber with near‐IR $\lambda_{0}^{*}$ when it is under strain, but this fiber is not visible after subtraction in the unstrained state, since its ground state Bragg reflection is outside the visible spectrum; only upon straining the fiber, λ_0_ gets blueshifted into the visible part of the spectrum, allowing the fiber to be seen with red and green colors in the right column when ϵ_↔_ > 0.

Although the CLCE polarization contrast reduces upon strain, as discussed above, the right‐handed reflection remains stronger up to ϵ_*zz*_ ≈ 1,^[^ ^15^ ^]^ and the subtraction of left‐ from right‐hand‐polarized images thus allows to reveal the mechanochromic response with excellent contrast. Note that the strain levels indicated on the left of the figure give the strain of the carrier fabric; the resulting strain of the fiber depends on how it has been sewn into the fabric.^[^ ^15^ ^]^ Figures S14 and S15, Supporting Information, show the behavior at intermediate strains and Movie S4, Supporting Information, shows the white fabric manually stretched in different directions. While we here demonstrate the principle only during strain sensing, we note that the absence of an LC–isotropic transition demonstrated above means that the circularly polarized CLCE Bragg reflection is active at all temperatures. The background subtraction is thus equally feasible also during thermochromic and thermomechanical operation of CLCE fibers.

#### 2.4.2 Visualizing Localized Strains in Knotted Fibers

The act of tying a knot is extremely important across all aspects of our lives, from the mundane task of tightening our shoe laces to a surgeon tying a suture. Understanding the strain and stress in a knot is a key factor in predicting failures of strings, ropes, or other knotted filaments, but the complex interplay of topology, elasticity, and friction and the local variations of curvature‐dependent strain render a theoretical analysis difficult.^[^ ^40^ ^]^ With our cylindrically symmetric CLCE fibers, we can experimentally visualize strain in knots, as was done for overhand/trefoil and figure‐eight knots by Patil et al. using mechanochromic fibers made by rolling stacks of alternating rubber types.^[^ ^40^ ^]^ The easy way in which our CLCE fibers are produced constitutes a significant advantage, the more cumbersome method of Patil et al. possibly explaining why they studied no more than two knots experimentally. Another advantage is the possibility of circular polarization‐enabled background subtraction demonstrated above.

**Figure 5:**
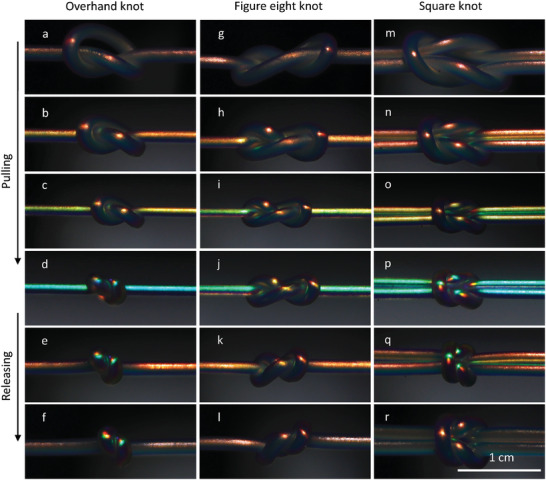
Mechanochromic strain visualization on different knots during tightening and relaxation; a–f) overhand knot, g–l) figure‐eight, and m–r) square (also called reef) knot.

In **Figure** **5**, we visualize the strain during tightening of three knots—overhand, figure‐eight and square or reef knot—as well as during relaxation afterward, using a CLCE fiber with red $\lambda_{0}^{*}$. Panels a/g/m show the fibers in the relaxed state, before tightening the knots. For small strain, a blueshift is seen only in the straight fiber sections while the knots retain their original color, see Figure 5b/h/n. Even when the straight segments have changed to yellow‐green color, the overhand and figure‐eight knots remain red, while the square knot shows a blue shift in the longitudinal, but not the transverse, sections, see panels c/i/o. The much delayed strain in the square knot compared to its external fiber segments contrasts with the simulations of Patil et al.,^[^ ^40^ ^]^ predicting a greater strain in the knot throughout the tightening. Most likely this is due to the self‐friction in our fiber being much greater than in the simulation (the square knot was not studied experimentally by Patil et al.).

Once the straight fiber acquires cyan color (ϵ_*zz*_ ≈ 1.3 according to Figure S5a, Supporting Information) all three knots change color, see Figure 5d/j/p. The overhand and square knots are cyan but the figure‐eight knot shifts only to yellow‐orange, revealing lower internal strain. As the fibers are relaxed, the overhand knot retains its high internal strain (e–f) while the figure‐eight (k and l) and square (q and r) knots revert to red, indicating negligible residual strain. Movies S5–S7, Supporting Information show the three experiments carried out on fibers with near‐IR $\lambda_{0}^{*}$ in real time.

## 3 Conclusions and Outlook

Using a dissolvable tube as template, we have produced cylindrically symmetric CLCE fibers with excellent radial helix alignment. The ground state pitch and corresponding retroreflection wavelength $\lambda_{0}^{*}$ are conveniently tuned from infrared to blue by changing the concentration of chiral dopant. The fibers show strong mechanochromic blueshift of more than ‐220 nm for 180% tensile strain, showing reflectance above the 50% of a relaxed cholesteric over most of the range. We explain this by the strain‐induced helix flattening, increasing reflectance at the cost of reduced circular polarization contrast. Nevertheless, the latter remains high enough to allow highlighting the mechanochromic response by subtracting images obtained through right‐ and left‐handed polarizers, removing the unpolarized background. We also demonstrate the ability of the CLCE fibers to visualize local fiber strain during tightening of knots and subsequent relaxation, revealing significant local variations depending on the knot type. The CLCE fibers exhibit monotonic thermochromic and thermomechanical response over a broad temperature range, confirming de Gennes' theoretical prediction that the LC–isotropic transition of cholesteric LCs is suppressed by crosslinking.

To scale up fiber production, the viscosity of the precursor should be minimized by working with monomer melts, possibly at elevated temperature, and an automated filling set‐up that exerts high pressure at the filling end while sucking vacuum at the opposite end should be highly useful. Moreover, to minimize waste and speed up the process, the templating tube should be tailormade as thin as possible for the purpose. By shifting to water‐soluble tubes made of, for example, polyvinylalcohol (PVA), water could replace the organic solvents for tube removal after CLCE crosslinking. Finally, an ideal approach to scale‐up is to switch to coaxial fiber spinning. We have demonstrated electrospinning of solid polymer sheath fibers with non‐polymeric cholesteric core^[^ ^19^ ^]^ or nematic LCE core,^[^ ^41^ ^]^ as well as microfluidic wet spinning of coaxial fibers with LC inside a rubber sheath.^[^ ^42^ ^]^ By adapting these methods to spin a dissolved polymer as sheath that will form a tube directly around a CLCE precursor core upon sheath solvent removal, our CLCE fibers could be made without length restriction. This is not a trivial route, however, as it is critical to balance minimal core–sheath interfacial tension against partial immiscibility,^[^ ^43^ ^]^ and also to avoid that the presence of the core reduces spinnability of the sheath.^[^ ^44^ ^]^ We are currently exploring these avenues and hope to report on the results in the near future.

## 4 Experimental Section

The oligomer synthesis was adopted from our previous work,^[^ ^13^, ^15^ ^]^ as detailed in Supporting Information. Dried oligomers were disolved in DCM and reactive chiral dopant (3R,3aS,6aS)‐hexahydrofuro[3,2‐b] furan‐3,6‐diyl bis(4‐(4‐((4‐(acryloyloxy)butoxy) carbonyloxy) benzoyloxy)benzoate) (LC756; 85 mg) (Synthon Chemicals) was added with different amount (generating different pitches; 3.5, 3, 2.8, and 2.65 wt% correspond to blue, green, red, and near‐IR $\lambda_{0}^{*}$). As photoinitiator, Irgacure 2022 (Sigma Aldrich) at a concentration of 2 wt% was used. The DCM concentration in this mixture was ≈60 wt%. The solution was stirred for 12 h and then dried until ≈20 wt% of DCM remained, yielding the solution that was filled into the tubular template.

For long fiber production, from monomers rather than oligomers, RM257 and EDDET were mixed at a mole ratio of 1.1:1, adding 2 wt% of the radical scavenger butylated hydroxytoluene, Irgacure2022 (2 wt%) and LC756 (4.3 wt% for green–blue $\lambda_{0}^{*}$), melting all components by heating to 80°C. To ensure a uniform mixture, this temperature was maintained while stirring for 2 h, after which it was cooled down to room temperature. The resulting mixture remained a uniform melt at room temperature at least long enough to complete all further steps without heating and without solvent addition. 0.5 wt% of dipropylamine (DPA) was now added, acting as catalyst for the first‐stage Michael addition reaction. For solvent‐free synthesis, DPA was added pure, but was sometimes diluted in DCM to maximize the accuracy in DPA concentration. In the latter case, the DCM concentration in the overall mixture was ≈10 wt%. After catalyst addition, the mixture was stirred for another 5 min before injecting into the tube.

For injecting the precursor into the tubular template (LDPE, inner diameter from 0.5 to 0.6 mm, wall thickness 0.15 mm, Scientific Commodities Inc., USA), a 2 mL syringe was filled with the monomer melt or the oligomer solution. The precursor was injected into the tube at a rate of 0.01–0.02 mL min^−1^ by means of a commercial syringe pump (KDS Legato 100, KD Scientific, USA). After filling, the tube was kept in an incubator at 45°C for 48 h to promote the CLC self‐assembly with radial helical structure, and to evaporate DCM when this was used. The successful CLC self‐assembly was recognized by the appearance of vivid colors through the LDPE tube, and its completion was determined by the color being uniform along the entire tube and stable in time. The samples were then transferred to an open‐top water bath and placed in a UV‐curing chamber (Opsytec Dr. Grbel Irradiation Chamber BSL‐01), allowing UV light irradiation from the top at wavelengths 330–450 nm and nominal UV light intensity of 200 mW cm^−2^ at the sample plane; the tubes were covered by 5 cm of water during the exposure. After 10 min UV exposure, the tubular LDPE template was dissolved by immersion of the samples into toluene (Sigma Aldrich) heated to 90°, for 30 min. This step was repeated three times with fresh toluene to remove all remaining LDPE. The CLCE fibers were then collected and dried in a vacuum oven at 150°C for 2 h to remove all toluene.

An Olympus BX51 polarizing optical microscope equipped with a digital camera (Olympus DP73) was used for microscopic characterization. Macroscopic images and videos were acquired with a Canon EOS 100D camera. SEM imaging was done using a JEOL JSM‐6010LA, operated in 5–20 kV range using an in‐lens secondary electron detector. Prior to SEM imaging, samples were coated with gold (≈3 nm thickness) using a Quorum Q150R ES coater. The reflection spectra were obtained using unpolarized white illumination and an Avantes AvaSpec‐2048 spectrophotometer, connected directly to a microscope. Reflection spectra were plotted using Origin Pro 9.1 (OriginLab) and the central retroreflection wavelength λ_0_ was obtained by fitting a single‐peak Lorentz function to each reflection peak in the raw data. A force gauge (Mark‐10, Model M3‐5) was used to characterize the mechanical properties. A microtome (Leica RM2200) was used to slice the CLCE fiber with 5 µm steps after it had been embedded in UV‐cured glue (Norland Optical Adhesive 160) for support. The diameter and length of the fibers were determined from the videos/images using image processing software Image J (version 1.53t, National Institutes of Health, USA). Blender (version 3.4.1) was used for creating 3D schematic illustrations of the director configurations in filaments/fibers in Figure 1c,d, and of the strain induced deformation process of the helical structure in Figure 2k and Figures S9 and S10, Supporting Information. In the later three figures, the positions and directions of all rods were determined according to the calculation in Section S6, Supporting Information. DSC was performed using a DSC823 (Mettler Toledo, USA) with a scanning rate of 10°C min^−1^ between −40 and 180 °C in a nitrogen atmosphere (9.4 mg CLCE fiber was used).

## Conflict of Interest

The authors declare no conflict of interest.

## Supporting information

Supporting Information

Supplemental Movie 1

Supplemental Movie 2

Supplemental Movie 3

Supplemental Movie 4

Supplemental Movie 5

Supplemental Movie 6

Supplemental Movie 7

## Data Availability

All raw data for this article is openly available at https://zenodo.org/record/7835358#.ZD0GyuxBzPZ.

## References

[1] Z. Liu , T. Zhu , J. Wang , Z. Zheng , Y. Li , J. Li , Y. Lai , Nano‐Micro Lett. 2022, 14, 61. 10.1007/s40820-022-00806-8 PMC8844338 35165824

[2] W. Sun , Z. Guo , Z. Yang , Y. Wu , W. Lan , Y. Liao , X. Wu , Y. Liu , Sensors 2022, 22, 7784. 36298135 10.3390/s22207784 PMC9607392

[3] A. Leber , B. Cholst , J. Sandt , N. Vogel , M. Kolle , Adv. Funct. Mater. 2019, 29, 1802629.

[4] H. Bai , S. Li , J. Barreiros , Y. Tu , C. R. Pollock , R. F. Shepherd , Science 2020, 370, 848. 33184214 10.1126/science.aba5504

[5] Q. Shi , J. Sun , C. Hou , Y. Li , Q. Zhang , H. Wang , Adv. Fiber Mater. 2019, 1, 3.

[6] S. Seymour , Fashionable Technology, The Intersection of Design, Fashion, and Technology, Springer, New York 2008.

[7] A. Yetisen , H. Qu , A. Manbachi , H. Butt , M. Dokmeci , J. Hinestroza , M. Skorobogatiy , A. Khademhosseini , S. Yun , ACS Nano 2016, 10, 3042. 26918485 10.1021/acsnano.5b08176

[8] J. M. Clough , C. Weder , S. Schrettl , Macromol. Rapid Commun. 2021, 42, 2000528. 10.1002/marc.202000528 33210385

[9] B. H. Miller , H. Liu , M. Kolle , Nat. Mater. 2022, 21, 1014. 35915162 10.1038/s41563-022-01318-x

[10] M. Kolle , A. Lethbridge , M. Kreysing , J. J. Baumberg , J. Aizenberg , P. Vukusic , Adv. Mater. 2013, 25, 2239. 23355069 10.1002/adma.201203529 PMC3652040

[11] M. Malekovic , M. Urann , U. Steiner , B. D. Wilts , M. Kolle , Adv. Opt. Mater. 2020, 8, 2000165.

[12] H. Finkelmann , S. T. Kim , A. Munoz , P. Palffy‐Muhoray , B. Taheri , Adv. Mater. 2001, 13, 1069.

[13] R. Kizhakidathazhath , Y. Geng , V. S. R. Jampani , C. Charni , A. Sharma , J. P. Lagerwall , Adv. Funct. Mater. 2020, 30, 1909537.

[14] S.‐U. Kim , Y.‐J. Lee , J. Liu , D. S. Kim , H. Wang , S. Yang , Nat. Mater. 2022, 21, 4146. 10.1038/s41563-021-01140-x 34580435

[15] Y. Geng , R. Kizhakidathazhath , J. P. Lagerwall , Nat. Mater. 2022, 21, 1441. 36175519 10.1038/s41563-022-01355-6 PMC9712110

[16] P.‐G. de Gennes , J. Prost , The Physics of Liquid Crystals, Clarendon Press, Oxford 1993.

[17] C. Schütz , J. R. Bruckner , C. Honorato‐Rios , Z. Tosheva , M. Anyfantakis , J. P. F. Lagerwall , Crystals 2020, 10, 199.

[18] H.‐S. Kitzerow , B. Liu , F. Xu , P. Crooker , Phys. Rev. E 1996, 54, 568. 10.1103/physreve.54.568 9965101

[19] E. Enz , J. Lagerwall , J. Mater. Chem. 2010, 20, 6866.

[20] M. Urbanski , C. G. Reyes , J. Noh , A. Sharma , Y. Geng , V. S. R. Jampani , J. P. Lagerwall , J. Phys. Condens. 2017, 29, 133003. 10.1088/1361-648X/aa5706 28199222

[21] M. O. Saed , A. H. Torbati , D. P. Nair , C. M. Yakacki , J. Visualized Exp. 2016, 107, e53546. 10.3791/53546 PMC4781659 26862925

[22] M. Warner , E. Terentjev , R. Meyer , Y. Mao , Phys. Rev. Lett. 2000, 85, 2320. 10978000 10.1103/PhysRevLett.85.2320

[23] P. A. Bermel , M. Warner , Phys. Rev. E 2001, 65, 010702. 10.1103/PhysRevE.65.010702 11800667

[24] P. Bermel , M. Warner , Phys. Rev. E 2002, 65, 056614. 10.1103/PhysRevE.65.056614 12059738

[25] P. Cicuta , A. Tajbakhsh , E. Terentjev , Phys. Rev. E 2004, 70, 011703. 10.1103/PhysRevE.70.011703 15324068

[26] P. Zhang , G. Zhou , L. T. de Haan , A. P. Schenning , Adv. Funct. Mater. 2021, 31, 2007887.

[27] J. Ma , Y. Yang , C. Valenzuela , X. Zhang , L. Wang , W. Feng , Angew. Chem., Int. Ed. 2022, 61, e202116219. 10.1002/anie.202116219 34962037

[28] T. White , D. Broer , Nat. Mater. 2015, 14, 1087. 26490216 10.1038/nmat4433

[29] C. Ohm , M. Brehmer , R. Zentel , Adv. Mater. 2010, 22, 3366. 20512812 10.1002/adma.200904059

[30] P.‐G. de Gennes , Phys. Lett. A 1969, 28, 725.

[31] D. J. Broer , G. N. Mol , Polym. Eng. Sci. 1991, 31, 625631.

[32] A. J. Kragt , N. C. Zuurbier , D. J. Broer , A. P. Schenning , ACS Appl. Mater. Interfaces 2019, 11, 28172. 31290319 10.1021/acsami.9b08827 PMC6689893

[33] Y.‐S. Zhang , S.‐A. Jiang , J.‐D. Lin , C.‐R. Lee , J. Mater. Chem. C 2020, 8, 5517.

[34] J. A. Sol , R. F. Douma , A. P. Schenning , M. G. Debije , Adv. Mater. Technol. 2022, 8, 2200970.

[35] Y.‐S. Zhang , H.‐S. Weng , S.‐A. Jiang , T.‐S. Mo , P.‐C. Yang , J.‐D. Lin , C.‐R. Lee , Adv. Opt. Mater. 2021, 9, 2100667.

[36] C. M. Yakacki , M. Saed , D. P. Nair , T. Gong , S. M. Reed , C. N. Bowman , RSC Adv. 2015, 5, 18997.

[37] M. Schwartz , Y. Geng , H. Agha , R. Kizhakidathazhath , D. Liu , G. Lenzini , J. P. F. Lagerwall , Multifunct. Mater. 2021, 4, 022002.

[38] Y. Geng , R. Kizhakidathazhath , J. P. Lagerwall , Adv. Funct. Mater. 2021, 31, 2100399.

[39] H. Agha , Y. Geng , X. Ma , D. I. Avşar , R. Kizhakidathazhath , Y.‐S. Zhang , A. Tourani , H. Bavle , J.‐L. Sanchez‐Lopez , H. Voos , M. Schwartz , J. P. F. Lagerwall , Light Sci. Appl. 2022, 11, 309. 36284089 10.1038/s41377-022-01002-4 PMC9592545

[40] V. P. Patil , J. D. Sandt , M. Kolle , J. Dunkel , Science 2020, 367, 71. 31896713 10.1126/science.aaz0135

[41] A. Sharma , J. P. Lagerwall , Materials 2018, 11, 393.29518917

[42] L. W. Honaker , S. Vats , M. Anyfantakis , J. P. F. Lagerwall , J. Mater. Chem. C 2019, 7, 1158811596.

[43] S. Vats , M. Anyfantakis , L. Honaker , F. Basoli , J. Lagerwall , Langmuir 2021, 37, 13265. 34735163 10.1021/acs.langmuir.1c01824 PMC8600680

[44] C. Reyes , J. Lagerwall , ACS Appl. Mater. Interfaces 2020, 12, 26566. 32420728 10.1021/acsami.0c03338 PMC7302509

