# Supplementary material for: Multiresponsive Cylindrically Symmetric Cholesteric Liquid Crystal Elastomer Fibers Templated by Tubular Confinement
Source: Adv Sci (Weinh). 2023 Apr 26;10(19):2301414. doi: 10.1002/advs.202301414 (PMC10323659; doi:10.1002/advs.202301414)
Supplement: Supplementary file 1 — Supporting Information [file ADVS-10-2301414-s003.pdf]

ADVANCED SCIENCE
Open Access

Supporting Information

for *Adv. Sci.*, DOI 10.1002/advs.202301414

Multiresponsive Cylindrically Symmetric Cholesteric Liquid Crystal Elastomer Fibers Templated by Tubular Confinement

*Yong Geng and Jan P.F. Lagerwall**

Supporting information

S1 Oligomer synthesis procedure

The oligomer synthesis was adopted from our previous work. [13, 15]. The monomers shown in Figure 1a, 1,4-bis-[4-(3-acryloyloxypropyloxy)benzoyloxy]-2-methylbenzene (RM257, 1.92 g) (Wilshire Technologies) and 2,2-(ethylenedioxy) diethanethiol (EDDET, 0.59 g) (Sigma Aldrich), were dissolved at a mole ratio of 1:1 in dichloromethane (DCM, 6 mL). Butylated hydroxy toluene (BHT; 5,3 mg, 0.2 wt.%) (Sigma Aldrich) was added as radical scavenger. Triethylamine (TEA, 1.6 ml) was used as a catalyst for the Michael addition reaction and the solution was stirred at room temperature for 24 h. The resulting mixture was washed thrice with 1M aqueous HCl to remove TEA, washed with brine solution, dried over $MgSO_4$ and then filtered. A rotary evaporator was used to remove the solvent.

S2 Characterization of CLCE structure orthogonal to the fiber axis

Figure S1 shows the same fiber cross section as in Figure 1k in the main paper, imaged using POM without analyzer (a), between crossed polarizers (b; this image is the same as in Figure 1k) and between crossed polarizers with a first-order λ-plate (optical path difference, OPD, 530 nm) inserted, respectively. Adding to the discussion in the main paper, we here note that the color in (b) changes from the center of the cross section, appearing with the pink color found at about 500 nm OPD in a Michel-Lévy chart, to the yellow of about 850 nm OPD at the perimeter. This color change reflects a gradual increase in Δn from core to perimeter, suggesting a corresponding increase in degree of liquid crystal orientational order along the radius.

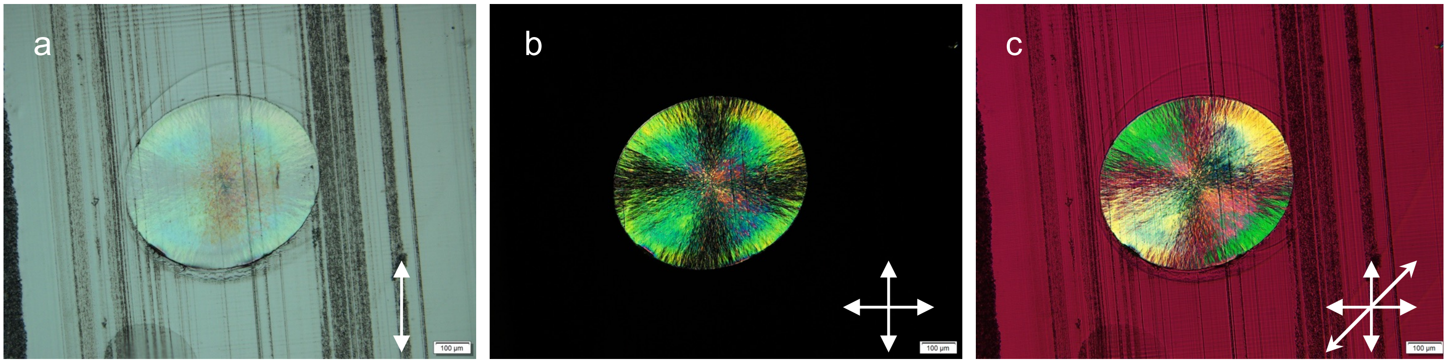

Figure S1: POM characterization of 5 μm thick CLCE fiber cross-section slice embedded in NOA glue; (a) transmission mode without analyzer; (b) between crossed polarizers; (c) between crossed polarizers with waveplate inserted with its optic axis 45° respect to polarizer and analyzer.

The conclusions concerning radial helix orientation and radially increasing order are further corroborated in (c), the introduction of the λ-plate yielding valuable complementary information. Throughout the top right and bottom left quadrants, we now see a color shift corresponding to a 530 nm move leftward in the Michel-Lévy chart compared to (b), effectively cancelling out the birefringence near the core, which now appears black in these quadrants between crossed polarizers. In the top left and bottom right quadrants, the color instead changes 530 nm to the right in the Michel-Lévy chart. Since the λ-plate has $\Delta n > 0$ with optic axis oriented in the SW–NE direction, this proves that the cross section of the CLCE with helically modulated director has $\Delta n_h < 0$ with radial optic axis. This is what we expect with this chemistry, for which $\Delta n_{nh} > 0$, since observation perpendicular to $\mathbf{m}$ of a CLCE with p short enough to give visible Bragg reflection reveals an effective optical behavior that is the result of averaging over the helical refractive index modulation. [17] This explains the observed macroscopic birefringence $\Delta n_h < 0$.

In **Figure S2**a–b we show the POM texture as the cross section is rotated around its symmetry axis between crossed polarizers without (a) and with (b) the first-order λ-plate inserted. There is no qualitative change, giving further evidence of excellent radial helix and cylindrical symmetry of the fiber from which the cross section was cut.

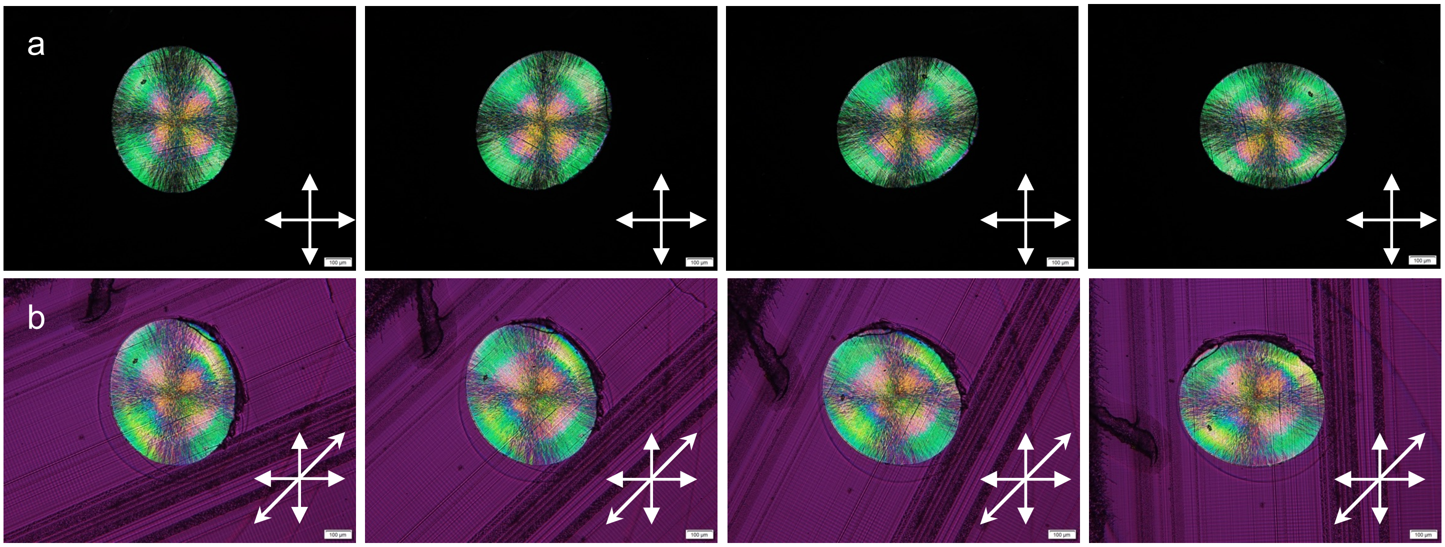

Figure S2: POM characterization of cross sections of the fiber in Figure S1 (a–b) in the main paper, upon rotation around the viewing axis, without (a) and with a first-order λ waveplate inserted (b). Scale bar is 100 μm.

S3 Reflection color as a function of viewing angle

The radial orientation of **m** turns the fiber into a retroreflector for any set of identical illumination and viewing directions perpendicular to the fiber, as shown in **Figure S3**a–e. We place an extended piece of fiber with red λ_0^* on a glass slide for investigation by POM as a function of the angle β by which the glass slide is tilted from the imaging plane. In one case the fiber is placed perpendicular to the tilting plane (Figure S3a), in the other parallel to it (Figure S3f). As shown in Figure S3b-e, the former configuration leads to constant apparent color at wavelength λ_0^* regardless of β, confirming the excellent radial orientation of **m** throughout the fiber. In the second configuration, the color also remains constant but it quickly loses intensity for increasing β (Figure S3g–j), the fiber now being tilted towards the viewing direction, thus losing the retroreflection ability.

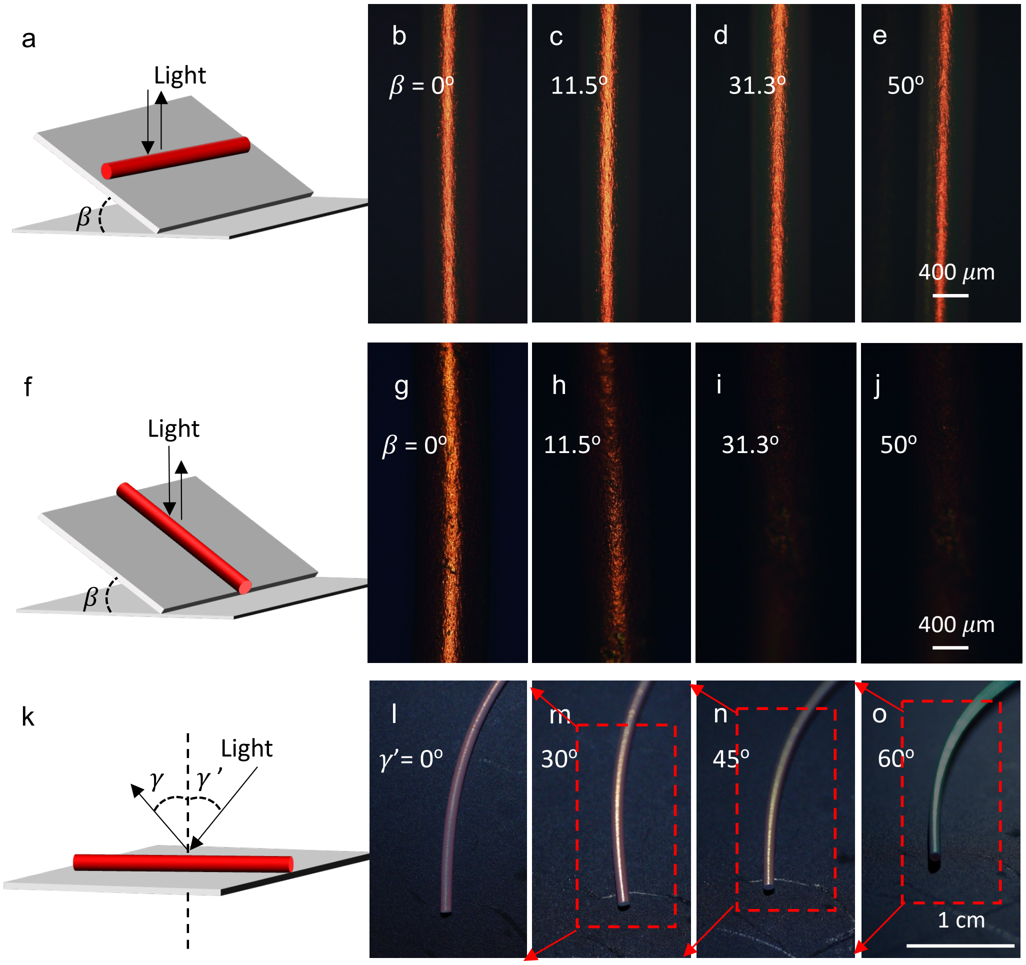

Figure S3: Retroreflection and specular reflection from a CLCE fiber with red λ_0^*. Drawings (a, f and k) schematically show the experiment configuration for each row, the fiber being placed on a glass slide in (a) and (k) which can be tilted by varying angles β. Panels (b–e) show the constant red retroreflection as the fiber is imaged along the illumination direction using a POM, from different angles β in a plane perpendicular to the fiber. Panels (g–j) show the corresponding appearance as the fiber is in the tilting plane, leading to loss of retroreflection for $\beta > 12°$. The bottom row shows the fiber photographed from a greater distance by a regular DSLR camera and imaged by a white light source oriented to give specular reflection into the camera, for various angles γ. The color now blueshifts as γ increases, in line with Eq. (1) along the top of the fiber, where $\gamma = \theta$.

To investigate the apparent color under specular reflection conditions, we do not use POM but instead a DSLR camera with macrolens and a white light source, both fixed on a goniometer set-up along a line parallel to the CLCE fiber, such that the macroscopic illumination angle γ' is always mirroring the imaging angle $\gamma = -\gamma'$, see Figure S3k. The angles γ and γ' are defined with respect to the horizontal black substrate on which the fiber is placed. The same fiber as before is used, but due to the greater imaging distance, we now see a large enough section to reveal that the fiber curves to the right at the top. The apparent color is now blue-shifted as $|\gamma|$ and $|\gamma'|$ increase, see Figure S3l–o. This is expected from Eq. (1) in the main paper, given that $\gamma = \theta$ along the top of the fiber.

S4 Mechanochromic response of fibers with varying λ_0^*

Figure S4 shows the observed color (a–i) and reflected spectra (j) of a fiber with red λ_0^* as a function of axial strain ϵ_{zz}. **Figure S5**a summarizes λ_0 as a function of ϵ_{zz} for three fibers with λ_0^* in the near-IR, red and yellow, respectively. Figure S5b shows the same results normalized to λ_0^*, clearly showing that the response to strain gets stronger as the relaxed-state pitch gets shorter.

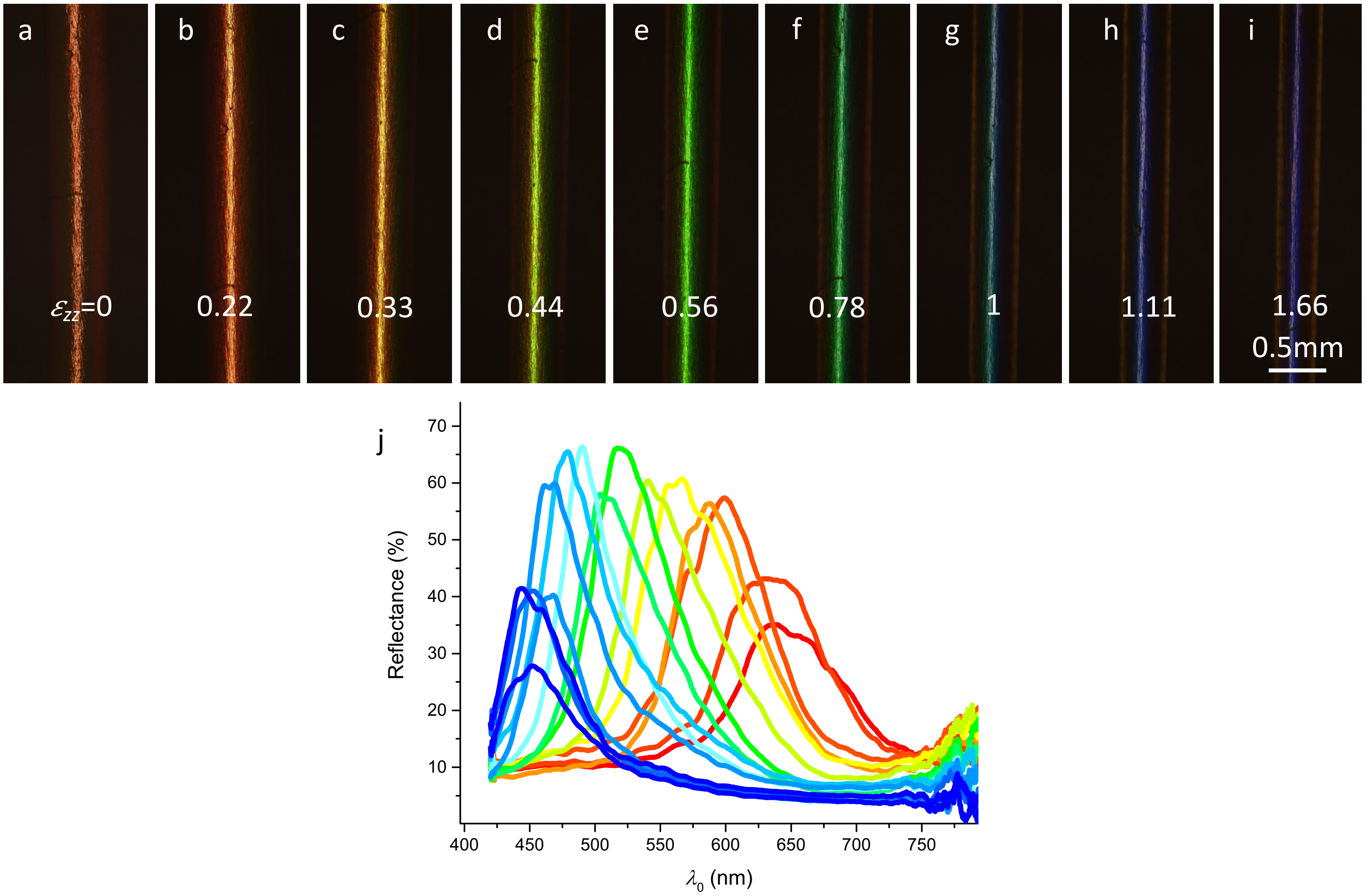

Figure S4: (a-i) Reflection-mode POM images and (b) spectra of a CLCE fiber with red λ_0^* under elongational strain.

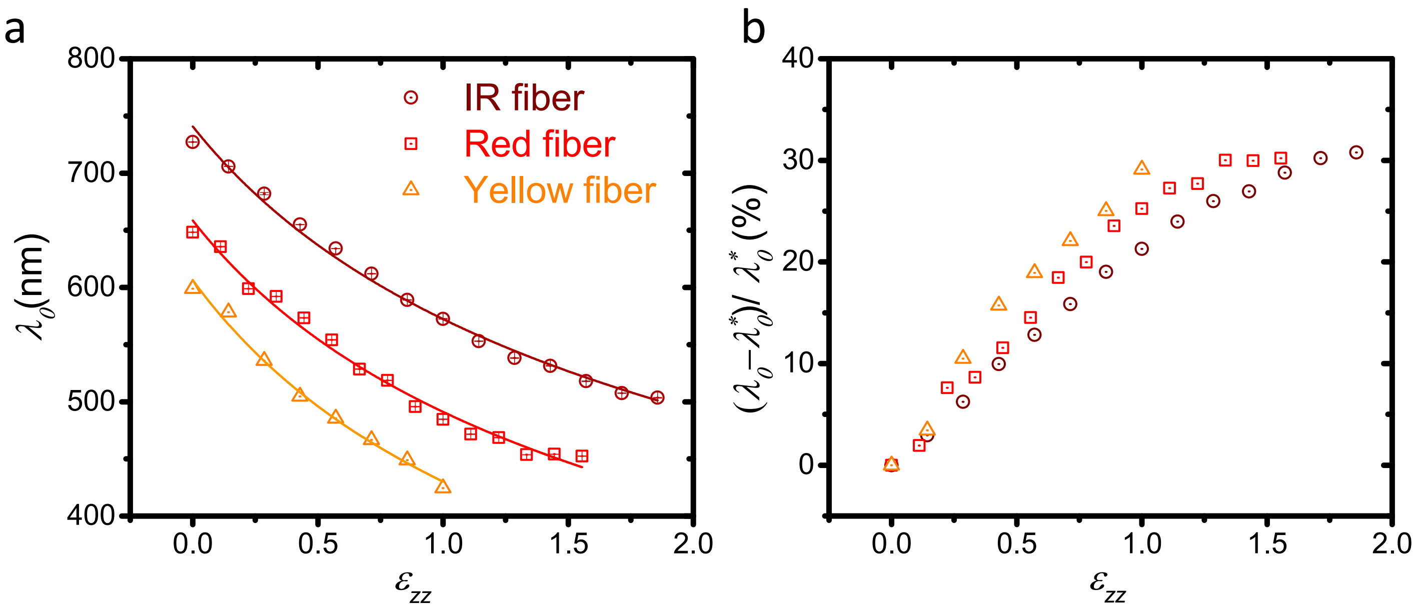

Figure S5: Retroreflection wavelength λ_0 (a) and the corresponding normalized wavelength shift (b) for three fibers with different relaxed-state color vs. axial fiber strain ϵ_{zz}. The solid lines in (a) show best fits of the power law $\lambda_0 = \lambda_0^*(1+\epsilon_{zz})^{-\nu}$, yielding the following numerical results; IR fiber: $\lambda_0^* = 741$ nm, $\nu = 0.37$; Red fiber: $\lambda_0^* = 659$ nm, $\nu = 0.42$; Yellow fiber: $\lambda_0^* = 606$ nm, $\nu = 0.5$.

By fitting Eq. (4) to the mechanochromic data, we find that all our fibers have ν closer to $\nu^{iso} = 0.5$ of isotropic rubbers than the $\nu^{xz} = 2/7$ obtained by Cicuta et al. for pitch compression along $\hat{x}$ of flat CLCEs elongated along $\hat{z}$, [25] which also fitted the behavior of our previous belt-like CLCEs very well. [15] As explained by Terentjev and co-workers, [25] the deviation from the isotropic rubber case is expected due to the soft elasticity of CLCEs, being tougher to compress along than perpendicular to $\mathbf{m}$. In the flat geometry, a volume-conserving behavior could be recovered by assuming a Poisson's ratio $\nu^{yz} = 5/7$ for compression perpendicular to the tensile strain and the helix, but this is not possible in our cylindrical case, hence a greater effective Poisson's ratio might be expected.

The increase in Poisson's ratio in our cylindrical fibers compared to flat CLCEs, further boosted by reducing λ_0^*, is highly attractive from the perspective of applying the CLCE fibers for strain monitoring, since it means a greater relative wavelength shift for the same strain level. We define the relative wavelength shift $\overline{\Delta\lambda}$ as:

$$\overline{\Delta\lambda} = \frac{\lambda_0^* - \lambda_0}{\lambda_0^*} = \frac{\lambda_0^*(1-(1+\epsilon_{zz})^{-\nu})}{\lambda_0^*} = 1-(1+\epsilon_{zz})^{-\nu} \tag{5}$$

where we used Equation (4) of the main paper in the second step. We can then calculate the percentual increase Δ in $\overline{\Delta\lambda}$ from increasing the Poisson's ratio from $\nu = 2/7$ to $\nu = 0.5$ as:

$$\Delta = 100(\frac{\overline{\Delta\lambda}(\nu=0.5)}{\overline{\Delta\lambda}(\nu=2/7)} - 1) = 100\left(\frac{1-(1+\epsilon_{zz})^{-0.5}}{1-(1+\epsilon_{zz})^{-2/7}} - 1\right) \tag{6}$$

The result for five different strain levels are tabulated in Table S1. We see that the dif-

Table S1: Percentual increase Δ in relative wavelength shift for five different levels of axial fiber strain ϵ_{zz}.

ϵ_{zz}	Δ
0.1	73%
0.5	68%
1.0	63%
1.5	60%
2.0	57%

ference can be quite significant, especially at small strains.

However, one must remember that the validity of our fitting procedure has its limitations, since Eq. (4) assumes constant ν. The actual value may well change with increasing ϵ_{zz}, as has been reported for NLCEs under strain. [45] The fact that the value of λ_0^* obtained from fitting is clearly larger than the measured value for each of the fiber types may be a hint that a complete analysis must allow strain-dependent values of the Poisson's ratios. Another explanation for this deviation may be incomplete relaxation after manipulating the fibers for the experiment, resulting in a residual compressive strain along $\hat{r}$ that gives an apparent relaxed-state λ_0 that is slightly less than the true λ_0^*.

S5 Mechanical testing of the CLCE fibers

To get an idea of the overall mechanical properties of the CLCE fibers, we subject a fiber with near-IR $\lambda_0^* \approx 726$ nm to repeated cycles of increasing strain while measuring the stress, following the program shown in **Figure S6**a. Fitting a linear function to the small-strain stress–strain curve, we obtain the initial Young's modulus value of $/Y_0 \approx 0.70$ MPa, see Figure S6b. Finally, we continue straining until fiber rupture, measuring the stress in the process, giving us a strain and stress at break of $\epsilon_{zz}^{max} = 2.93$ and $\sigma_{zz}^{max} = 52.3$ MPa, respectively, see Figure S6c.

stress.pdf

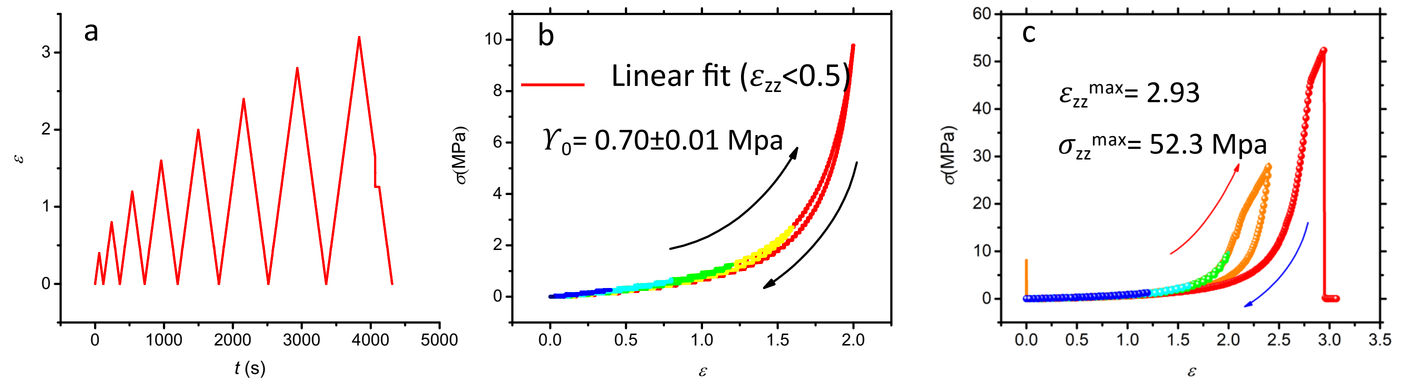

Figure S6: Strain stress curve with different maximum strain repetition. (a) The program of strain vs. time during stress-strain measurements. (b) Strain-stress cycles with low maximum strain, where linear fitting was performed to obtain the initial Young's modulus. (c) Stress-strain with higher maximum levels until fiber rupture.

S6 Visualization of helix unwinding process using Warner–Terentjev theory

The Warner and Terentjev teams developed the theory for describing CLCE helix unwinding, [22, 25] resulting in the following equation for the angle ϕ of $\mathbf{n}$ with respect to the tensile direction $\hat{z}$ as a function of strain ϵ_{zz}, with the helix along $\hat{x}$ (we have rotated the coordinate system such that the z-axis is identical to that of our cylindrical coordinate system): [25]

$$\tan 2\phi = \frac{2(1+\varepsilon_{zz})^{1/4}(r-1)\sin(2\tilde{q}z)}{(r-1)((1+\varepsilon_{zz})^2+(1+\varepsilon_{zz})^{-3/2})\cos(2\tilde{q}z)+(r+1)((1+\varepsilon_{zz})^2-(1+\varepsilon_{zz})^{-3/2})}. \quad (7)$$

Here r is an intrinsic measure of local polymer chain anisotropy and $\tilde{q} = \frac{2\pi}{p}(1+\epsilon_{zz})^{1/4}$ is a reduced wave vector representing the helical modulation under strain.
Solving Eq. 7 for ϕ with respect to the distance z along the fiber normalized by $\tilde{q}$ for several selected levels of strain ϵ_{zz}, adding or subtracting π when required to appropriately deal with the degeneracy of the $\tan 2\phi$ function, we obtain the diagram in **Figure S7**. Having access to ϕ we can visualize the projection of $\mathbf{n}$ into the xy and yz-planes, respectively, by taking $\sin\phi$ and $\cos\phi$, respectively, see **Figure S8**.

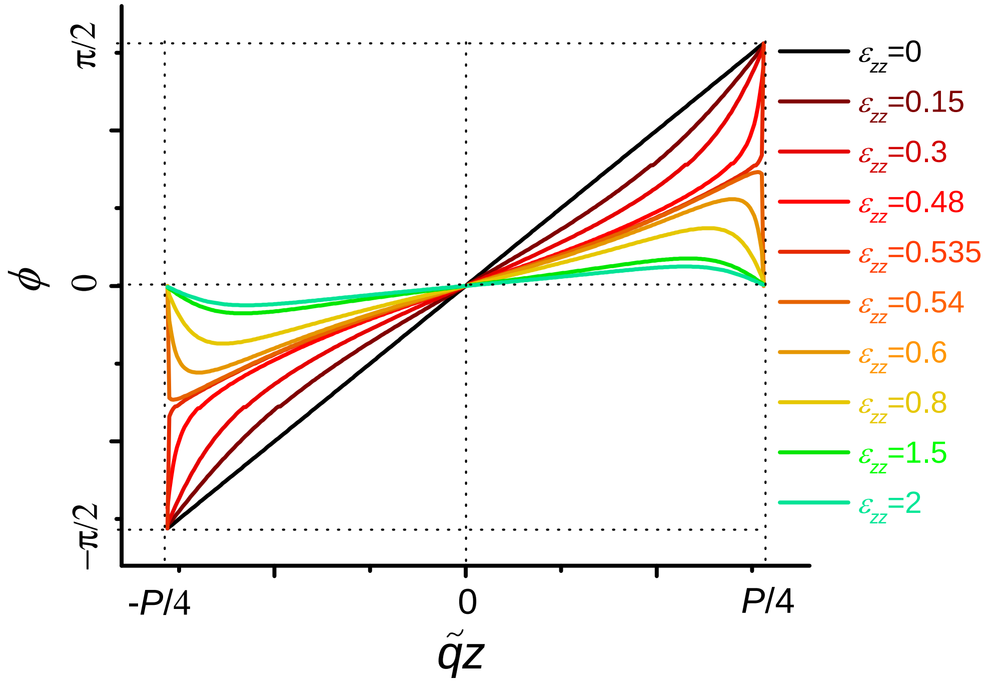

Figure S7: Calculated director angle ϕ around the helical axis under different strain from Eq. 7. To match the experimental data (see main paper), we have set $r = 5.4$, giving a critical strain ϵ^c_{zz} between 0.535 and 0.54.

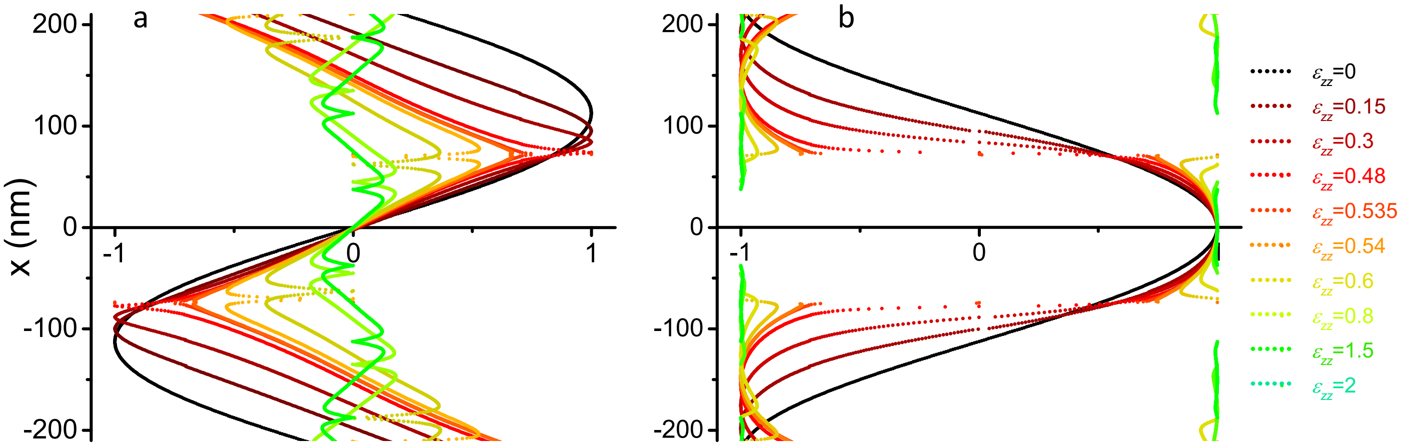

Figure S8: Director projection (a) perpendicular and (b) parallel to the strain direction, obtained by plotting $\sin\phi$ and $\cos\phi$, respectively, with ϕ from Fig. S7.

To better visualize the helix unwinding process, we render several 3D views (using Blender) of 72 rods evenly distributed along one helix pitch, each rod representing the 'local optic axis' (at this scale, orders of magnitude smaller than light wavelengths, a true optic axis cannot be defined), extending from $-\mathbf{n}$ to $+\mathbf{n}$. We do so for multiple strain levels, the orientations of each rod extracted from the data in Figure S7, viewed along three different directions in **Figure S9**. Two of these views are reproduced in Figure 2k in the main paper. We also replicate the structure at each strain level along multiple radii of a circle, yielding the schematic illustrations of the director field throughout the fiber cross section shown in **Figure S10**. The drawings are highly idealized and not to scale: here the radius is one pitch length, whereas the real fibers have radii on the order of 250 μm while the pitch is on the order of 0.4 μm (corresponding to red retroreflection with an average refractive index of about 1.5), i.e., the real radius is on the order of 600 pitch lengths.

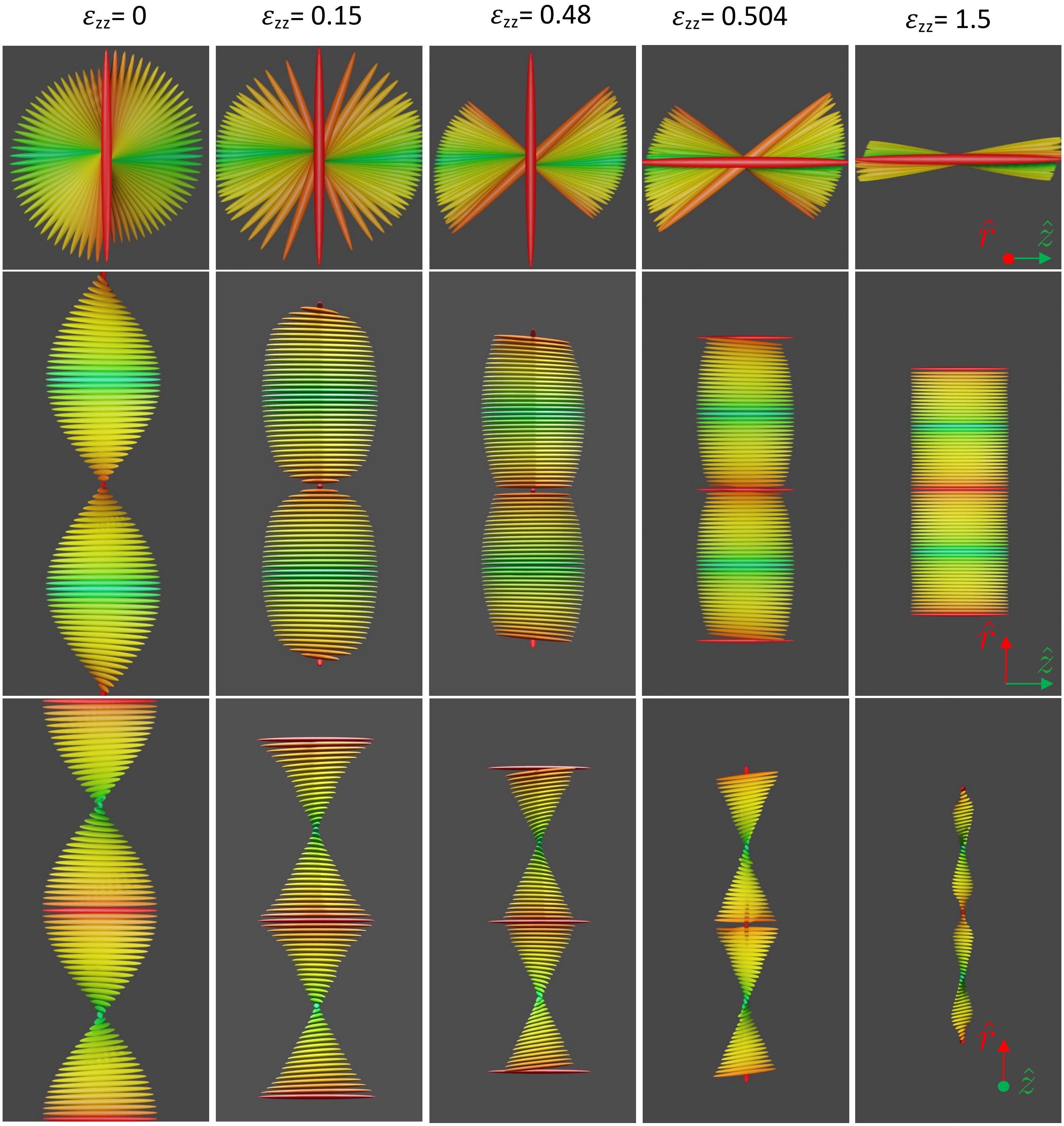

Figure S9: Helical structure deformation process under various axial strain levels ϵ_{zz} illustrated by drawing multiple rods representing the 'local optic axis', extending from **-n** to +**n**, along one pitch. The three rows represent different viewing directions, as identified by the coordinate system at the bottom right of each row.

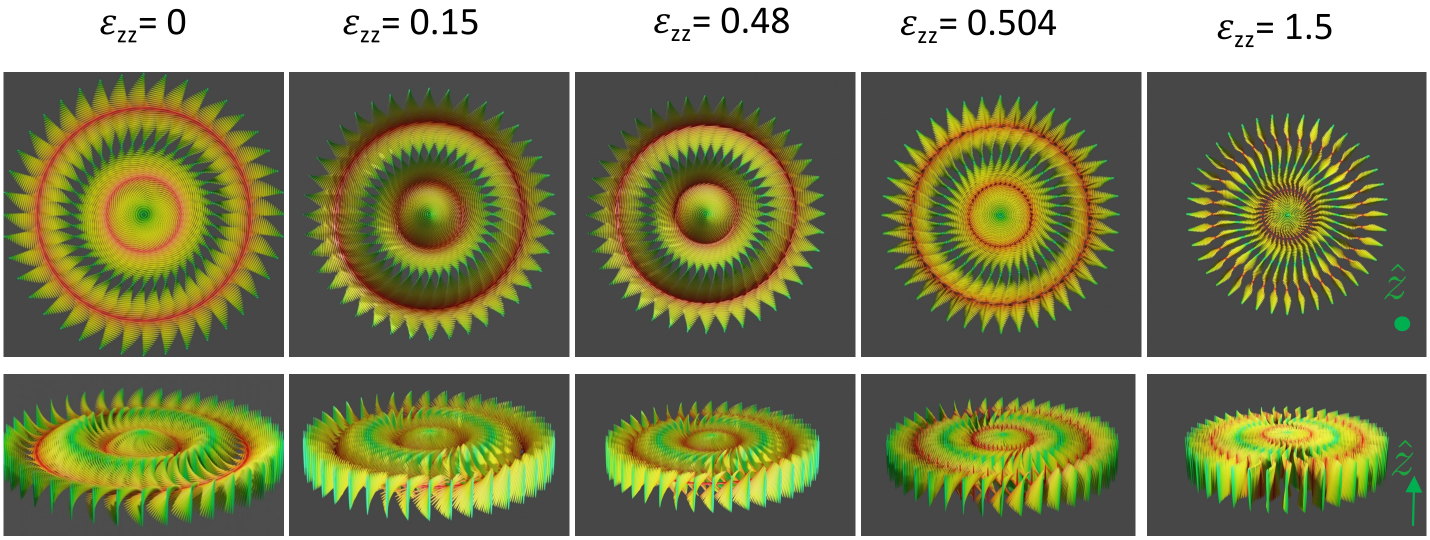

Figure S10: Schematic illustration of the director configuration in a cross-section of the CLCE fiber under various axial strain levels ϵ_{zz}, achieved by radial replication of the calculated structures in Figure S9 within a circle. For simplicity, only a single pitch has been been used to complete one full radius.

S7 Response to temperature variation

Figure S11 shows the reflection color of the fiber with green retroreflection in the relaxed state at room temperature, as it is heated from 20°C to 170°C and then cooled back. An excerpt of these images was included in Figure 3 in the main paper.

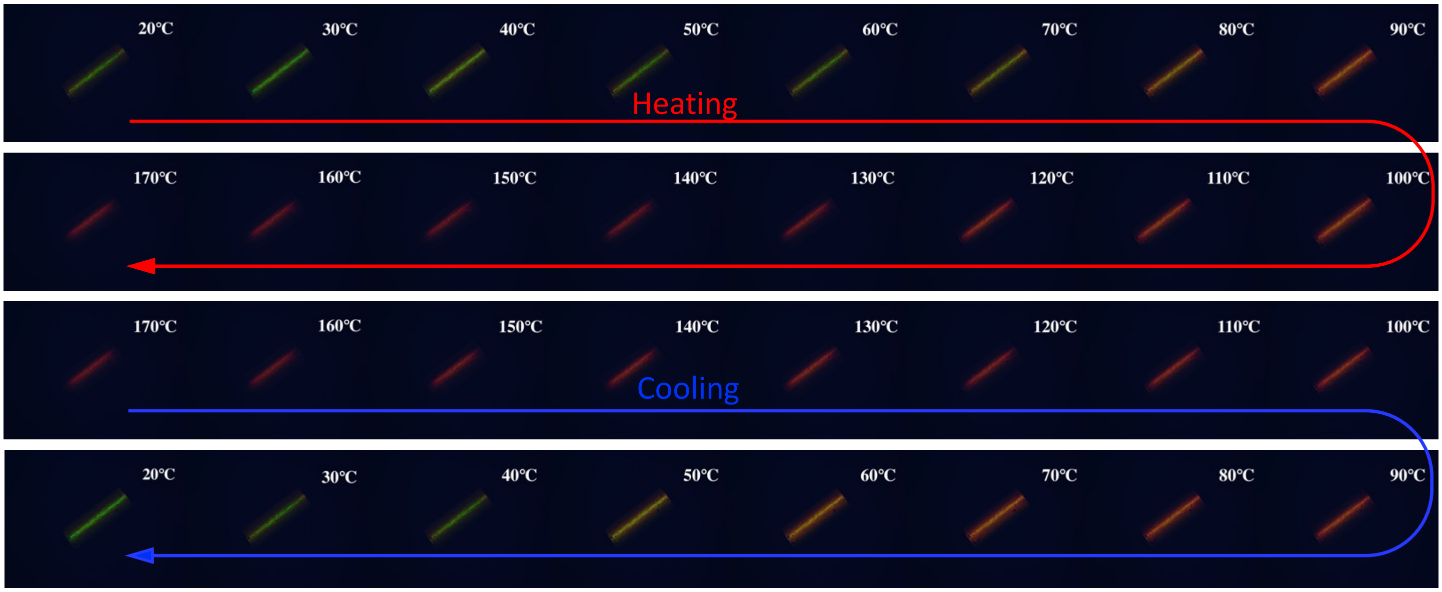

Figure S11: Full set of reflection-mode POM images of a piece of CLCE fiber with green λ_0^* at room temperature during heating and cooling process with a rate of 5°C/min from 20 to 170°C and back.

Figure S12 shows the relative radial expansion versus the relative length compression for the fiber with green λ_0^* as it is heated. The data are the same as those in Figure 3f in the main paper.

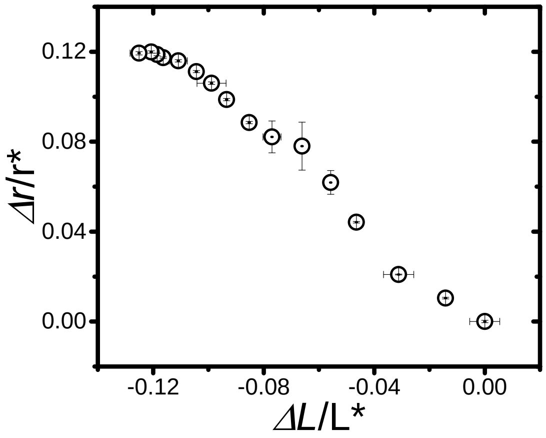

Figure S12: Relative radius change vs. relative length change of a green retroreflection fiber during heating process.

Figure S13 shows the thermogram of a fiber with green λ_0^* as obtained by differential scanning calorimetry (DSC). The fiber had been kept in a vacuum oven at 150°C for 2 hours prior to the experiment, and a small hole was poked into the DSC pan in order

to let the air surrounding the fiber be replaced by nitrogen during the first run, and to avoid excess pressures from building up in the pan during heating. The response during first cooling is not shown, as the air had not yet been replaced by nitrogen and as the first cooling was done at a very fast cooling rate (not defined by the operator) from room temperature to the program starting point at -40°C. The first cycle followed on this initial cooling and the second cycle was identical to the first. The response during these two cycles is perfectly identical, as seen by the curves overlapping. We see the glass transition at $T_g \approx 0$°C but no further transition can be seen at higher temperatures, confirming de Gennes' expectation that crosslinking the helical structure of a cholesteric suppresses the clearing transition.

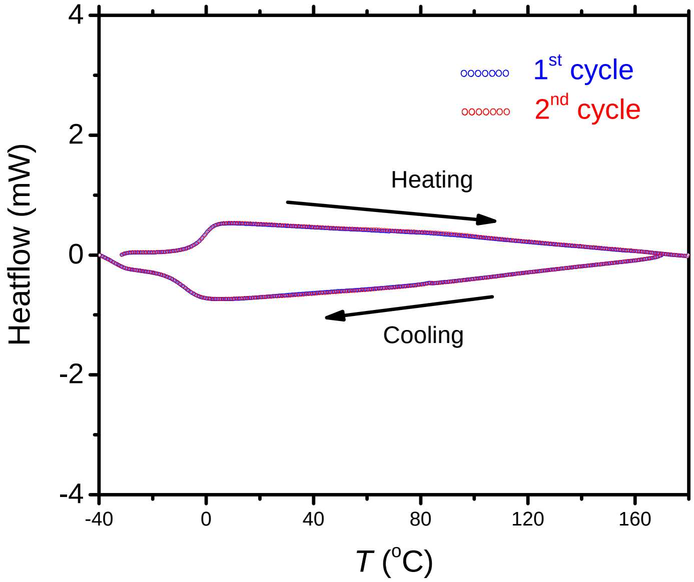

Figure S13: Thermogram from differential scanning calorimetry (DSC) of a fiber with green λ_0^* upon two consecutive heating and cooling runs. The heating and cooling rates were both 10 K/min.

S8 Background subtraction at various strain levels

Figures S14 and S15 show the response of the two samples studied in Figure 4 in the main paper, adding several intermediate strain levels. These examples were obtained by taking the two photos one after the other, switching polarizer in between (the action changing the overall illumination somewhat, leading to some background remaining after subtraction), and then doing the subtraction afterwards in an image processing software (ImageJ), but this can easily be done in real-time with a simple hardware consisting of a beam splitter, two cameras and two polarizers, as described and demonstrated by Agha et al. [39] Beyond the mechanochromic strain sensing, this enhanced detection of CLCE fibers embedded into clothing can be very useful in reading critical information hidden in environments with strong visual pollution or localizing persons wearing fabrics labeled with CLCE fibers. [37–39] For instance, if life vests were decorated with CLCE fibers, search and rescue operations at sea might be greatly facilitated by subtracting the background of the vast non-circularly polarized ocean.

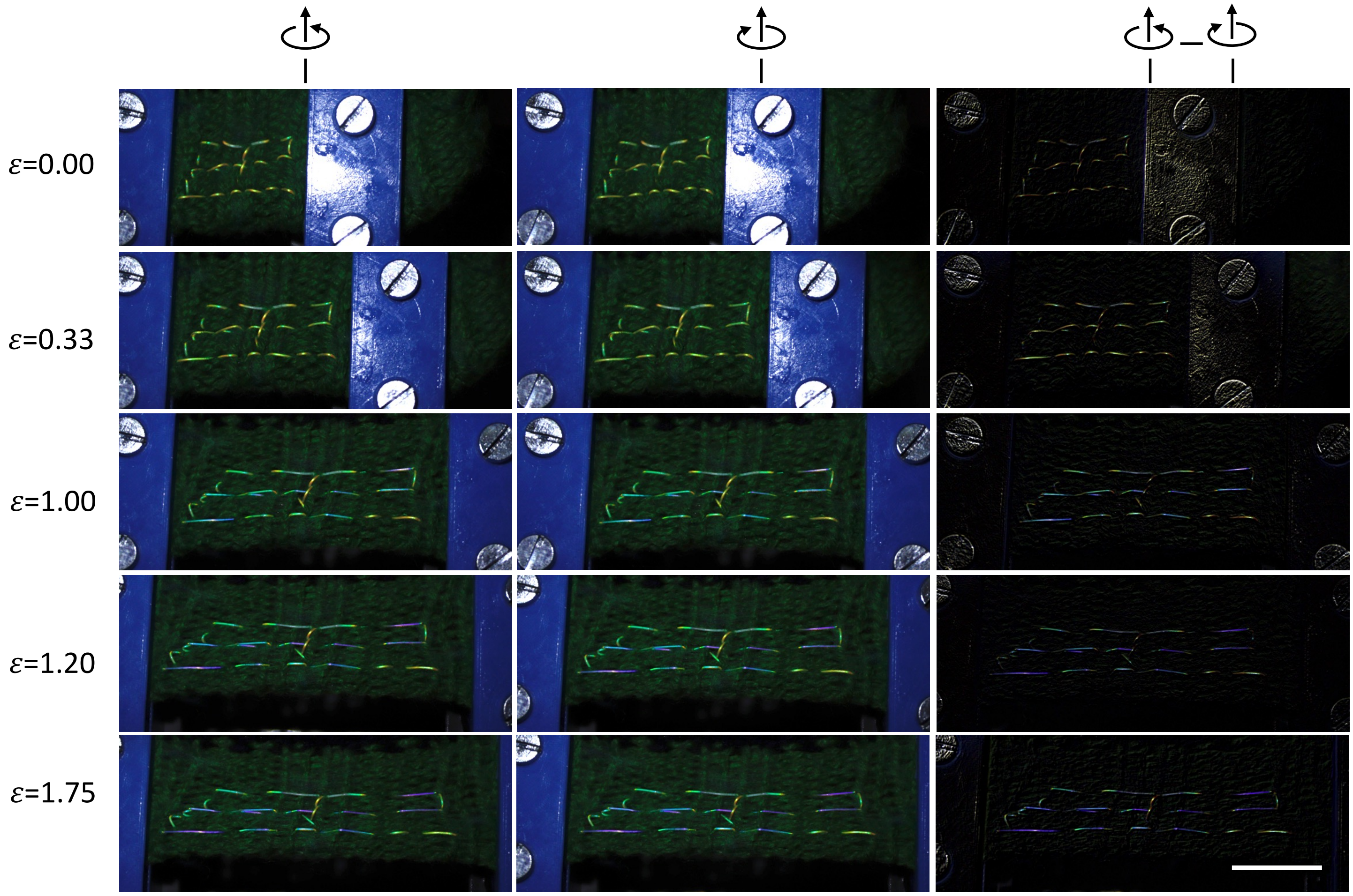

Figure S14: Background subtraction during straining of a fiber with green λ_0^* (Scale bar is 1 cm)

.

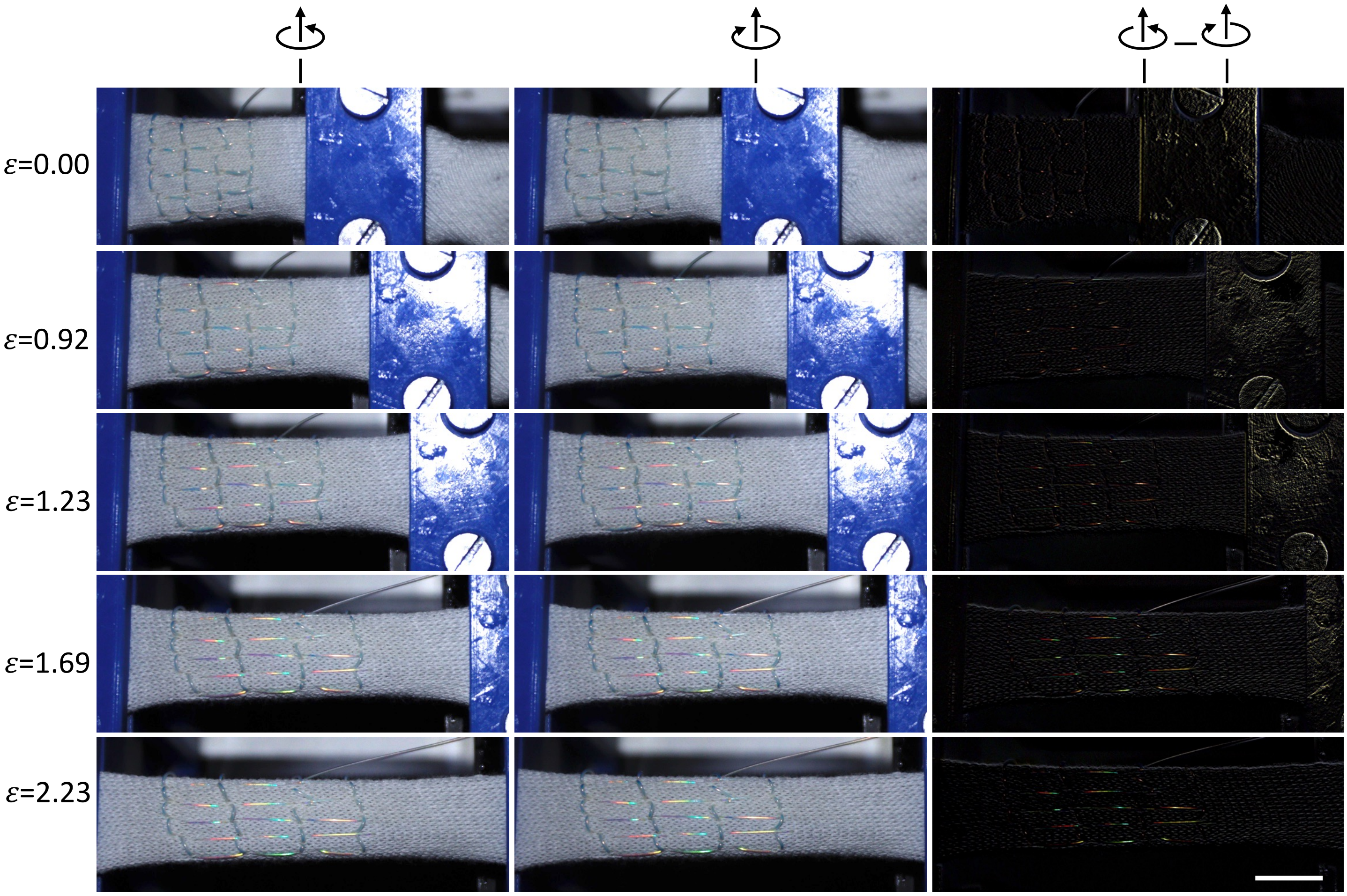

Figure S15: Background subtraction during straining of a fiber with near-IR λ_0^* (Scale bar is 1 cm)

.
